# Pathways to inclusion: A scoping review exploring equity, diversity, and inclusion in mental health and addictions patient navigation programs

**DOI:** 10.1371/journal.pmen.0000557

**Published:** 2026-06-25

**Authors:** Amy K. W. Cho, Ruchika Suri, Christina Cutler, Anthony J. Levitt, Roula Markoulakis

**Affiliations:** 1 Sunnybrook Research Institute, Toronto, Ontario, Canada; 2 Sunnybrook Health Sciences Centre, Toronto, Ontario, Canada; 3 Department of Psychiatry, University of Toronto, Toronto, Ontario, Canada; 4 Department of Occupational Science and Occupational Therapy, University of Toronto, Toronto, Ontario, Canada; 5 Rehabilitation Sciences Institute, University of Toronto, Toronto, Ontario, Canada; PLOS: Public Library of Science, UNITED KINGDOM OF GREAT BRITAIN AND NORTHERN IRELAND

## Abstract

Mental health and addictions (MHA) patient navigation programs have gained traction in recent years, helping clients overcome barriers and access suitable resources. Equity-deserving communities (EDCs) continue to experience significant disparities in MHA outcomes, due to additional systemic barriers to care. This scoping review explores the approaches and practices utilized by MHA patient navigation programs to support the unique needs of EDCs. A search was conducted in four electronic databases. Articles were required to be focused on MHA patient navigation services and EDCs (including low-income individuals, racial and ethnic minorities, members of 2SLGBTQI+ communities, people with disabilities, Indigenous Peoples, and newcomers), peer-reviewed studies, written in English, and available as full-text. Three members of the research team conducted title, abstract, and full-text screening. Extracted data underwent descriptive synthesis and thematic analysis. The database search identified 1382 unique studies, 40 of which remained for full-text review after title and abstract screening. A total of 16 studies were extracted. Four themes emerged surrounding considerations and approaches needed to appropriately support EDCs in MHA patient navigation: barriers to equitable access to MHA services, influences on equitable service utilization, supports provided by MHA navigation to address complex needs, and design and delivery of inclusive MHA navigation programs. These themes encompass important considerations for MHA navigation programs when supporting EDCs to improve reach, engagement, and outcomes. This review provides crucial information regarding equitable navigation approaches when supporting EDCs with MHA concerns, including addressing specific barriers, leveraging facilitators, and practicing cultural competence. The findings enhance the current understanding of how known benefits of MHA patient navigation services can be maximized for marginalized populations, thereby supporting advancements toward health equity.

## 1. Introduction

### 1.1. Mental health and addictions issues

Mental health and addictions (MHA) issues represent significant contributors to the global burden of disease [1]. Despite rising prevalence rates, only 6.9% of those affected by MHA issues worldwide are receiving effective treatment [2, 3]. This disparity is attributed to various intersecting factors, with patients often being burdened by financial, emotional, time, geographical, and informational barriers to care [4, 5]. In particular, several studies have reported disproportionate rates of MHA concerns and lack of access to MHA services among historically marginalized communities who experience additional systemic barriers [2,6–8].

### 1.2. Equity-deserving communities

Equity-deserving communities (EDCs) are groups who face unjust barriers, based on their social identities, that limit equal access to education, employment, health care, and other opportunities [9]. Many social identities, such as income, race, gender identity, ability status, and citizenship status, can intersect to further shape experiences of power and oppression. This review focuses on groups known to face significant barriers in the healthcare system, including low-income individuals, racial and ethnic minorities, members of 2SLGBTQI+ communities, people with disabilities, Indigenous Peoples, and newcomers [9,10].

EDCs are affected by major inequities in MHA care and patient outcomes worldwide [2]. Systemic discrimination, exclusion, and a lack of culturally appropriate supports exacerbate the negative health outcomes seen disproportionately among these groups [5,11–14]. For example, a study by Farahi and Cénat [15] found that anxiety symptoms were 6.94 times more likely among participants who experienced very high levels of racial discrimination compared to those who experienced lower levels of racial discrimination. Kingsbury et al. [16] found that the risk of suicide was 7.6 times higher among transgender adolescents, who are more likely to experience minority stress and consequently poorer mental health, compared to cisgender heterosexual adolescents. The findings of these studies highlight how the added daily challenges faced by individuals belonging to EDCs impact their mental well-being. Despite the disparate rates of MHA concerns among EDCs, these groups remain less likely to access MHA services and are more likely to receive lower quality care compared to non-equity deserving groups [17–19]. For instance, a study by Manning et al. [20] reported provider bias to be a major barrier to accessing mental healthcare among nearly 70% of people living with disabilities. Sundareswaran et al. [21] identified language discordance as a hurdle to care for newcomers, including when booking appointments over the phone, understanding medical vocabulary, and accessing translated written documents. Newcomers’ mental healthcare experiences are directly impacted by the lack of funding for and absence of adequate professional interpretation services [22]. Healthcare funding schemes further disadvantage low-income and uninsured individuals, for whom high out-of-pocket costs for certain mental health services or prescription medications create additional stress and uncertainty [21,23,24]. Competing priorities, such as employment responsibilities, also often take precedence over seeking care [21,22,25]. Colonial policies continue to diminish Indigenous health needs through the underdevelopment and underfunding of health centers in remote communities and on reserves. As a result, the far distances to medical facilities and limited infrastructure make it challenging to receive timely care [26]. Ultimately, structural and systemic barriers perpetuate cycles of untreated MHA needs and worsening conditions in equity-deserving populations [17,18]. It is evident that appropriate supports are urgently needed to close the gap in MHA care and outcomes.

### 1.3. Mental health and addictions navigation services

Patient or system navigation (referred to simply as “navigation” throughout) is a community-based healthcare delivery intervention aimed at reducing barriers to timely diagnosis and/or treatment. The first described navigation program was implemented by Dr. Harold Freeman in 1990 in response to critical issues surrounding access to breast cancer care for Black women of lower economic status [4]. This program made dramatic strides in advancing health equity, increasing the five-year breast cancer survival rate from 39% to 70% by ensuring timely medical care [4]. Navigation services have expanded widely since to support patients with identifying appropriate supports in complex care systems for a variety of conditions (e.g., dementia [27], diabetes [28], HIV [29]). In the MHA system, the multitude of available services and prominent issues of care fragmentation often make it challenging for patients to determine the best care pathway [30]. Numerous MHA navigation programs have consequently emerged in recent years, providing patients with credible up-to-date information on services, connecting them to suitable resources, and empowering them to exercise autonomy over their own health [31–33]. Barrier reduction, client-centered support, and integration of care are core themes seen across MHA navigation programs [31]. There are various models of care used, including professional navigation by clinicians or trained staff and lay navigation by peers. Delivery methods offered may include virtual (i.e., email, text message, phone), in-person, or a combination of both [31,32].

The evolution of navigation services has brought with it a growing base of scientific literature [34]. Studies assessing MHA navigation have demonstrated the substantial promise of these programs in improving care experiences, participation, and outcomes (e.g., reduced number of mental and physical health problems, improved quality of life) [32]. However, recent reviews have been largely focused on cancer care [35,36]. For EDCs who face unique barriers in the healthcare system, the approaches used in MHA navigation may require particular consideration. There has been no scoping review conducted to date synthesizing the application of MHA navigation services to best support individuals belonging to EDCs. This is needed to determine how such programs can be developed and improved to alleviate barriers to appropriate care and better serve the needs of these communities. A comprehensive review to map the literature on how existing MHA navigation services specifically serve EDCs is therefore a crucial step in the process of developing more responsive and effective services, and in so doing, promote greater health equity.

## 2. Methods

A scoping review was conducted to thoroughly examine existing literature on strategies to support EDCs in MHA navigation programs. Methodological guidelines from Arksey & O’Malley and the Joanna Briggs Institute were followed [37,38]. A study protocol was developed *a priori* and registered on Open Science Framework on July 10, 2025 (https://osf.io/ucb6m). The Preferred Reporting Items for Systematic Reviews and Meta-Analyses extension for Scoping Reviews (PRISMA-ScR) checklist was applied to ensure good reporting (S1 File) [39].

### 2.1. Research question

This scoping review aimed to answer the research question: *what are the approaches and practices of MHA navigation programs to support EDCs, including low-income individuals, racial and ethnic minorities, members of 2SLGBTQI+ communities, people with disabilities, Indigenous Peoples, and newcomers?*

### 2.2. Eligibility criteria

Articles were included if they were focused on MHA navigation services, to ensure that information was relevant to the research question. Similarly, articles were included if they were focused on EDCs. Based on the aims of this review and existing literature that has identified groups facing unjust barriers, EDCs were defined as low-income individuals, racial and ethnic minorities, members of 2SLGBTQI+ communities, people with disabilities, Indigenous Peoples, and newcomers [9,10]. Articles were required to be peer-reviewed to ensure evidence-based information. Only articles written in English were included to ensure feasibility and an accurate understanding.

Articles that discussed services not primarily focused on navigation or MHA were excluded to maintain relevance to the topic of interest. Articles that were not fully focused on an EDC defined in this study (e.g., findings pertaining to EDCs reported as a sub-group analysis rather than being the focus of the study) were also excluded. Additionally, conference abstracts, protocols, dissertations/theses, and book chapters were excluded. Lastly, articles were excluded if full-text access was unavailable, given that a thorough review of content would not be possible.

### 2.3. Search strategy

The search strategy was developed by a research librarian at Sunnybrook Health Sciences Centre (CC). The search drew from several existing search filters and literature reviews. Some filters were left unchanged: poverty and social class [40], and newcomers [41]. Some filters were altered for inclusivity: sexual minorities [42], Indigeneity [43], and disability [44]. Filters for ethnicity and racialized people, MHA, and patient navigation were original. The search was run on July 4, 2025 in the electronic databases MEDLINE (Ovid), Embase (Ovid), CINAHL (EBSCOHost), and APA PsycINFO (Ovid). Key terms used to develop the search include: 1) equity-deserving communities (e.g., “sexual and gender minorities”, “racial groups”, “Indigenous Peoples”) AND 2) mental health (e.g., “mental disorders”, “substance use”, “addiction”) AND 3) navigation programs (e.g., “patient navigation”, “navigation services”). No date limits or platform-specific filters were used to ensure a comprehensive search. The full search strategy for each database can be found in the supplemental files (S2 File).

### 2.4. Study selection

All identified articles were imported into Covidence for de-duplication and screening. The inclusion and exclusion criteria were carefully applied to decide on whether a study should be included. Only studies meeting all inclusion criteria were included.

Two independent reviewers (AC, RS) first conducted a pilot title and abstract screening with 20 articles, with each reviewer using a structured Excel spreadsheet to indicate each study’s citation details and the reason for inclusion or exclusion. A discussion was then held to reach consensus on conflicts based on comparison of Excel spreadsheets and ensure reviewers had an aligned understanding of the eligibility criteria before continuation of the screening process. Following this, the remaining titles and abstracts were screened by two reviewers (AC, RS), with a third reviewer (RM) available to resolve conflicts throughout. After completion of title and abstract screening, two team members (AC, RS) conducted a pilot full-text review with five articles. Each reviewer again used a structured Excel spreadsheet to indicate each study’s citation details and the reason for inclusion or exclusion. A discussion was held to reach consensus before the remaining full-texts were reviewed by the two team members (AC, RS). A third reviewer (RM) resolved any conflicts that arose.

### 2.5. Data extraction

An extraction tool was developed in Microsoft Excel, with review and revision by the study investigator (RM) for appropriateness. One reviewer (AC) charted the included studies and the study investigator (RM) regularly checked for accuracy. Extracted data included Covidence reference number, title, authors, publication year, country, study objectives, study design, types of EDCs included, intervention details, strategies to support EDCs, patient outcomes, and lessons learned. This charted information was used to descriptively and numerically summarize the sources.

### 2.6. Data synthesis

The charted data was thematically analyzed in MAXQDA. MAXQDA is a software program used for coding in qualitative data analysis [45]. This software supported the visualization and identification of key data in the included studies, which allowed for synthesis into the findings of this review.

Two team members (AC, RS) first independently coded five segments to create an initial codebook. A discussion was held with the study investigator (RM) to refine this codebook, which was then used for the remaining data. Patterns in codes were identified to develop themes and sub-themes that responded to the research question.

### 2.7. Positionality statement

The authors recognize that positionality shapes the research process. The research team comprised members with different social identities, lived experiences, and professional backgrounds relevant to the populations of focus in this review. Representation of diverse genders, racial and ethnic backgrounds, ability statuses, and ages ensured that the search, study screening, and data extraction steps were informed by a range of perspectives.

## 3. Results

**Fig 1:**
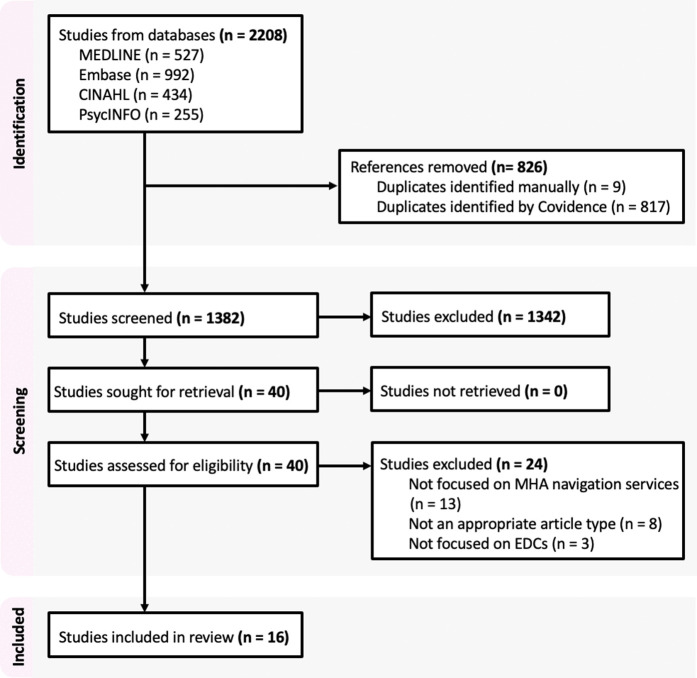
PRISMA-ScR study selection flow diagram.

The database search identified 2208 studies, 826 of which were identified as duplicates and removed. The remaining 1382 studies underwent title and abstract screening. A total of 40 studies met the inclusion criteria and were retrieved for full-text review. Finally, 16 studies were extracted. The PRISMA-ScR flowchart detailing study inclusion can be seen in Fig 1.

### 3.1. Description of included studies

#### 3.1.1. Publication year & country of article origin.

Publication year of the included studies ranged from 2014 to 2025, with most being published in 2023 (n = 4). The lack of studies from before 2014 reflect the expansion of patient navigation over recent years. All studies originated from the United States (n = 16) (see Table 1).

**Table 1:** Reviewed articles’ study design, objective, and EDCs included (ordered alphabetically by author).

Author (Year), Country	Study Design/Objective	MHA Concern of Focus	EDCs of Focus
Corrigan et al. (2014), United States	Narrative review Examine literature on peer navigators to improve integrated care for ethnic minorities with severe mental illness.	Serious mental illness (unspecified)	Ethnic minorities
Corrigan et al. (2017), United States	Mixed methods study Identify healthcare needs, barriers, and solutions using findings from a community-based participatory study.	Serious mental illness (defined as individuals who cannot live independently or work due to psychiatric disorders, such as personality disorders or major affective disorders)	Ethnic minorities
Eliacin et al. (2021), United States	Mixed methods study Conduct a pre-implementation evaluation while developing a peer-led navigation program to examine views of interest holders and important considerations for adoption in mental health care.	Mental illness and substance use disorder (unspecified)	Racial minorities
Eliacin et al. (2023a), United States	Randomized controlled trial Examine study design feasibility and the acceptability and preliminary effects of a peer-led navigation program.	Mental illness (participants had mood, stress, psychotic, and personality disorders) and substance use disorder	Racial minorities
Eliacin et al. (2023b), United States	Qualitative study Assess participants’ experiences of a peer-led navigation program and views of components that influence communication with clinicians.	Mental illness (participants had mood, stress, psychotic, and personality disorders) and substance use disorder	Racial minorities
Giraldo et al. (2024), United States	Retrospective chart review (observational study) Examine impacts of peer support on retention in an outpatient clinic, and assess differences across racial and ethnic groups to determine intersectional factors that may influence retention in care.	Opioid use disorder	Low-income individuals belonging to racial and ethnic minority groups
Koob et al. (2024), United States	Retrospective observational study Assess the impact of patient-level factors, community-level factors, and social drivers on access to pediatric mental health services in a navigation program.	Mental and behavioral health needs (unspecified)	Racial minorities, low-income individuals
Lasser et al. (2017), United States	Randomized clinical trial Assess the effects of a patient navigation and financial incentive intervention on smoking cessation.	Cigarette smoking	Low-income individuals, racial and ethnic minorities
Poleshuck et al. (2019), United States	Randomized comparative effectiveness trial Determine patient satisfaction, compare outcomes, and identify those likely to benefit from Enhanced Screening and Referral (ESR) versus Personalized Support for Progress (PSP) interventions.	Depression	Racial minorities, low-income individuals
Quintiliani et al. (2019), United States	Exploratory observational process evaluation study Evaluate the association between smoking-related and sociodemographic characteristics with levels of patient navigator delivered smoking cessation counseling receipt.	Cigarette smoking	Racial minorities, low-income individuals
Quintiliani et al. (2021), United States	Randomized pilot study Assess a patient navigation tobacco treatment intervention’s feasibility and acceptability.	Cigarette smoking	Racial minorities, low-income individuals
Running Bear et al. (2023), United States	Randomized controlled trial Determine effectiveness of an intervention (motivational interviewing with patient navigation) for improving alcohol treatment transition rates after detoxification and preventing detoxification readmission in 12-months. Evaluate patient navigation’s cost-benefit and cost-effectiveness.	Substance use (alcohol or drugs)	Indigenous Peoples
Sheehan et al. (2018), United States	Qualitative study Examine a peer navigator program’s strengths and weaknesses, focusing on the perspectives of service providers and service users.	Serious mental illness (unspecified)	Ethnic minorities
Silverstein et al. (2017), United States	Randomized pilot trial Test delivery of a patient navigation intervention in the Head Start preschool setting and obtain key parameter empirical estimates from controls.	Depression	Low-income individuals
van der Star et al. (2025), United States	Mixed methods study Describe integration of patient navigation with the Safety Planning Intervention (SPI), and examine the acceptability and feasibility of research procedures and intervention implementation.	Suicidality	2SLGBTQI+ members, racial minorities
Vance et al. (2023), United States	Open pilot trial Examine disparities in service use among Black youth in a suicide care coordination intervention.	Self-injurious thoughts and/or behavior	Racial minorities

#### 3.1.2. Study design.

All articles were peer-reviewed studies (n = 16), including randomized trials (n = 6), observational studies (n = 3), mixed methods studies (n = 3), qualitative studies (n = 2), an open pilot trial (n = 1), and a narrative review (n = 1) (see Table 1). All 16 sources in this review examined MHA patient navigation interventions for equity-deserving populations (see Table 2).

**Table 2:** Key findings from included articles (organized alphabetically by author).

Source	Key Findings and Strategies to Improve MHA Navigation for EDCs
Corrigan et al. (2014)	• Including peer navigators in programming can allow EDCs to feel better understood and supported. Peer navigators can better relate to and understand the unique challenges faced by patients belonging to minority ethnic groups (e.g., cultural concerns, family concerns). The authors found that peer navigation improved rates of prolonged treatment regimen completion, rehospitalization, inpatient days, and disruptions in care for patients of minority ethnic groups.
Corrigan et al. (2017)	• Citizenship/documentation status presents a barrier to healthcare engagement due to fear of deportation. Latino participants also expressed cultural stigma surrounding use of mental healthcare. Other challenges include language difficulties, lack of insurance, transportation barriers, employment barriers, poor awareness of illness, and unfamiliarity with mental health systems. Suggested solutions include offering peer navigators and multilingual service providers, providing culturally adapted services, ensuring service providers are aware of documentation/immigration challenges and confidentiality needs, engaging family members, and supporting pragmatic barriers (e.g., transportation, need for childcare). • Peer navigators should be trained in critical principles (e.g., being accepting, empowering), how to work in a team, fundamental skills to work with clients and respond to concerns (e.g., shared decision making, active listening, cultural competence, trauma informed care), skills in role management (e.g., maintaining boundaries), and available resources in the area.
Eliacin et al. (2021)	• Participants noted that the proposed peer-led patient navigation program’s focus on Veterans belonging to racial minority groups was divisive and may reduce social cohesion. Ineffective patient-provider communication was reported to be a major factor contributing to Veterans’ dissatisfaction with mental healthcare. • Implementing a peer navigator program may improve trust and satisfaction with mental health services. In clinics hesitant to utilize peer workers, it is important to spend time creating an accepting environment for the intervention, such as by engaging leadership to facilitate integration and secure resources.
Eliacin et al. (2023a)	• Participants receiving peer-led patient navigation showed statistically significant improvements in depression scores, mental health function, and satisfaction with the mental health clinic compared to controls. Peer navigation may improve mental health service engagement among minority groups. • Suggested modifications to peer-led patient navigation to improve session completion rates include offering multiple contact methods (e.g. virtual and in-person), more flexible session schedules, and greater peer coverage.
Eliacin et al. (2023b)	• Navigators’ limited availability was reported to be the main cause of program dissatisfaction among participants. Participants also identified the need for multiple navigation delivery methods. Gender concordance with peer navigators was another prominent recommendation, especially among females who had previously experienced sexual assault. Reported barriers reducing intervention satisfaction include navigators’ limited availability. • Minority veterans expressed appreciation for peer navigators who consistently communicated openly, supported their treatment adherence and patient-clinician communication, and had similar sociocultural backgrounds. Racial concordance was noted to be an important, positive programming feature. Positive experiences with the peer-led patient navigation program were attributed to peer navigators’ characteristics, including non-judgmental attitude, ability to engage diverse patients, and relatability.
Giraldo et al. (2024)	• Social stigma and negative views of treatments are major barriers to engagement with care among racial/ethnic minorities. Recovery peer navigators (RPNs) were shown to improve retention in care among racial and ethnic minority groups. RPNs’ positive effects may be related to their shared experiences and ability to provide psychosocial support. Incorporating RPNs in clinics can help mitigate the stigma felt by patients and ensure the provision of personalized support, thereby overcoming socioeconomic factors that affect care retention. • The location of first contact by the navigator may alter their engagement with patients and requires further investigation. For example, RPNs were found to be beneficial in the linkage and retention of patients with medications for opioid use disorder after treatment initiation from the carceral and emergency room settings. When referrals to RPNs were made through primary care, engagement with outpatient opioid use disorder treatment increased and acute care utilization decreased.
Koob et al. (2024)	• Standardized training and navigation protocols for meeting unique needs may enhance evaluation across systems, improve patient outcomes, account for social barriers, and optimize referral-to-service connection. • To provide tailored support to youth who experience more social barriers to care, navigation programs can integrate zip-code level social determinant of health estimates into referral-to-service connection suggestions. Youth residing in areas with comparatively higher “risk” levels and who may therefore face additional challenges (e.g., housing instability, food insecurity) should be provided additional support. This risk stratification can also help inform treatment plans. • Use of an integrated database (e.g. REDCap) across navigators can allow for streamlined data collection, merging of electronic health records and referral data, appropriate management of referrals, and reduced errors in manual data entry. Implementation of a closed-loop referral system may support improved communication of data between navigators and clinicians, reducing fragmented care experiences and increasing efficiency of navigator workflow.
Lasser et al. (2017)	• Participants who received a navigation and incentive intervention showed significantly increased smoking cessation rates compared to controls. • Race concordance between patients and navigators improves care experiences.
Poleshuck et al. (2019)	• Including decision aids can allow patients to feel better informed, strengthening competence and sense of autonomy. Providing patients with opportunities to voice their needs to an accessible and safe navigator may offer relief from depression symptoms and improve engagement in treatment/resources. • Participants expressed a desire for longer-term contact with navigators. Extending the length of the intervention period may be more beneficial for improving depression outcomes.
Quintiliani et al. (2019)	• Ensuring racial/ ethnic concordance with navigators may improve intervention receptiveness among patients.
Quintiliani et al. (2021)	• Difficulty reaching patients, such as due to disconnected phones, was a barrier to navigation delivery. Identifying appropriate communication pathways, such as having in-person contact at the hospital or providing patients with cell phones at discharge, may improve engagement with navigation services. Text messaging is another potentially effective navigation delivery method that should be further examined. • Navigation should be offered across clinical settings with and without inpatient treatment to improve reach. Social needs screening and navigation can occur during hospitalization.
Running Bear et al. (2023)	• One-on-one patient navigation can improve continuity of care for Alaska Native and American Indian (AN/AI) detoxification patients. Readmission is prevented by matching personal needs to suitable resources in the community.
Sheehan et al. (2018)	• Participants noted the need for more contact/time with navigators and more comprehensive resource offerings (i.e., expanding the list of services navigators can refer clients to). • The peer relationship, navigator accessibility, and trust built with the navigator were stated to be important factors. Participants highly endorsed the emotional support provided by navigators, which included enhancement of positive emotions. Navigators’ role in supporting problem-solving (e.g., complex appointments, difficult paperwork) and facilitating communication with providers was also reported as helpful. • Navigators noted barriers including difficulties with client engagement, need for more organizational support, and financial and insurance barriers to care for clients. Individuals who are undocumented face substantial challenges in accessing services, even with navigation. Additional training can help navigators with engaging difficult clients, managing emotions, and supporting social needs (e.g., employment).
Silverstein et al. (2017)	• ‘Homework’ sheets were developed by navigators to help ensure completion of follow-up tasks by study participants. To provide more time for psychoeducation and address time management concerns, the engagement session (open-ended questioning where navigators identified appropriate referrals by engaging clients in shared decision making) was extended from one in-person meeting to two in-person meetings. Navigators also ensured to address mothers’ prior dissatisfaction with mental health services. • Navigators were lay people (no prior formal mental health experience) who attended a two-day training to develop skills in cultural competency, community health work, patient advocacy, working with individuals with depression, organization, and mental health emergencies. Participants endorsed navigators as being respectful, dependable, able to identify factors affecting healthcare access, and able to listen. Navigation was suggested to be acceptable and feasible to support engagement in care among low-income mothers with depression.
van der Star et al. (2025)	• The navigation intervention effectively prevented new suicidal behaviors among youth belonging to sexual and gender minority groups. Participants found navigation to be helpful with identifying resources and scheduling appointments. Shared identities between patients and navigators, emotional and instrumental support provided by navigators, resources being catered to patient needs, and navigation flexibility were positive aspects of the intervention. Offering different communication options is valuable for patient engagement with navigation services. • Baseline assessment in initial appointments should be short in length.
Vance et al. (2023)	• Long-term service coordination and frequent engagement/contact may improve access to care. Black youth showed disparities in medication use, which may reflect a lack of trust in providers and/or limited information about treatments. Care coordinators reduced misconceptions about pharmaceutical treatments by encouraging use of community resources, reviewing formal/informal sources of support, and providing psychosocial education and support. • The intervention effectively reduced disparities in service use among Black youth. Suicide care coordination must consider patients’ treatment preferences and prioritize culturally responsive care, including integrating diverse supportive networks, psychoeducation, and strategies to overcome systemic/environmental barriers.

#### 3.1.3. EDCs in included studies.

Studies often included two or more EDCs (n = 7). Racial and ethnic minorities were most commonly included (n = 14), followed by low-income individuals (n = 7), Indigenous Peoples (n = 1), and members of 2SLGBTQI+ communities (n = 1) (see Table 1).

### 3.2. Identified themes

Four themes emerged from the included articles following analysis of MHA navigation intervention details, strategies to support EDCs, outcomes, and lessons learned. Themes included: barriers to equitable access to MHA services, influences on equitable service utilization, supports provided by MHA navigation to address complex needs, and design and delivery of inclusive MHA navigation programs. Each theme encompasses key considerations for MHA navigation programs when working with EDCs.

#### 3.2.1. Barriers to equitable access to MHA services.

The included studies emphasized three categories of barriers hindering EDCs’ access to MHA services: practical barriers, scheduling conflicts, and systemic barriers.

Practical barriersPractical barriers impacting access to care among EDCs include transportation difficulties, financial struggles, and privacy concerns. EDCs noted that the ability to travel to and from MHA services presented a critical barrier to receiving appropriate care, underscoring the need for transportation support [46–48]. Difficulty affording MHA services, which often require costly out-of-pocket payments, was also commonly discussed as a factor limiting care accessibility [47,49]. In addition, a perceived lack of privacy and concerns about confidentiality were reported to hinder EDCs from seeking MHA support [48].

***Scheduling conflicts.*** Scheduling conflicts discussed involve service availability and time management. EDCs reported that a major barrier to care was the lack of timely services, often due to providers’ and specialists’ limited availability, resulting in long waiting periods without support [47,49]. Difficulty managing time conflicts with other commitments, such as not being able to take time off work and challenges obtaining childcare, was another factor described to affect access to MHA services [46,48].

***Systemic barriers.*** Systemic barriers refer to the policies, practices, and behaviors embedded in the structures of organizations or social systems that unfairly disadvantage certain groups [50]. Those prominently affecting EDCs in the reviewed studies include language barriers, religion-related barriers, stigma, and citizenship-related barriers. Lack of language concordance with providers was noted to be a factor impacting service access. EDCs expressed the desire for multilingual providers who could speak their first language to better address cultural factors influencing their mental health [46]. In some religions, there is a particular stigma surrounding the pursuit of mental healthcare, where some believe that men are expected to not need such services or that the church will provide sufficient support instead [46,51]. Beyond religion, societal stigma and self-stigma around mental illness was reported to interfere with use of services and adherence to treatment [48,52-54]. Citizenship status was reported as another critical barrier to care, with undocumented individuals often avoiding mental health supports for fear of deportation. Undocumented individuals often lack insurance benefits, intersecting with other financial struggles to hinder access to costly MHA services [46,51].

***Barriers to accessing navigation services***. Barriers limiting accessibility of navigation programs include technology-related challenges. Delivery of navigation via phone was noted to be a challenge for participants without reliable access to a phone or who preferred in-person contact [55]. For service providers, inability to reach participants and disconnected phones was a barrier to navigation delivery [56].

#### 3.2.2. Influences on equitable service utilization.

The reviewed studies identified various barriers and facilitators that influence engagement with MHA services and navigation programs. In contrast to the first theme of “Barriers to equitable access to MHA services”, factors in this second theme limit or promote an individual’s continued use of a service/program after it has been initially accessed.

***Barriers and facilitators to MHA service utilization*.** A history of risky behaviors and the presence of mood disorders were factors found to act as barriers to MHA services [49]. However, having access to additional sources of support, such as family members or community resources, improves engagement with care [49]. Similarly, having a positive outlook can act as a facilitator to consistent utilization of MHA services [49]. When care is linguistically accessible (i.e., providers speak patients’ first language) and culturally consistent, EDCs were reported to be more likely to remain engaged with the service [46,49].

***Barriers and facilitators to navigation program utilization***. A major barrier to engagement with navigation programs echoed across many of the studies is navigators’ limited availability [51,57]. For example, participants in a peer-led navigation intervention expressed dissatisfaction with the program, attributed to difficulties coordinating sessions with their navigator [55,57]. In another navigation program for ethnic minorities, participants reported experiencing periods in which they unexpectedly did not hear from their navigator or that phone calls with their navigator gradually became more infrequent. The importance of regular interaction with navigators was stressed, with communication lapses impacting the patient-navigator relationship [51]. Difficulties communicating MHA needs with navigators act as another barrier to the continued use of navigation services [57,58]. Moreover, a history of risky behaviors, presence of mood disorders, or past suicide attempts were found to limit engagement with navigation programs [49]. However, as with engagement in MHA care, having additional supports and a positive outlook facilitates continued use of navigation services [49]. The ability of navigators to regulate their own feelings and be emotionally present further enhances continued use of navigation services, with EDCs feeling a greater sense of trust in their navigator [46]. For instance, peer navigators who previously overcame MHA issues may experience interpersonal, cognitive, and/or emotional challenges that affect the consistency of navigation provided. Studies stress sources of support (e.g., supervisors, debrief sessions) and emotional regulation training as critical resources for navigators working with EDCs [52]. Additionally, EDCs expressed that it would have been helpful for peer navigators to share more of their own emotions and struggles; this could have improved rapport building and relatability [51]. Leveraging service use facilitators also support continued engagement in navigation programs among EDCs. “Service use facilitators” refer to protective factors that can amplify EDCs’ coping abilities, internal strengths, and intentions for treatment [49].

#### 3.2.3. Supports provided by MHA navigation to address complex needs.

To meet the unique challenges faced by EDCs, navigators in the reviewed studies supported access to social programming [46–48,51], provided instrumental supports [46,51,57,58], engaged circles of support [46,47,49,51,57–59], delivered psychological supports [49,51,52,58,60], and offered supports that optimize the patient experience [48,49,51–53,55,57,58,61].

***Navigation support for access to social programming***. Social programming support provided by navigators included assistance accessing community-based and federal food assistance programs, overcoming housing instability, determining accessible methods of transportation to MHA appointments, and identifying appropriate childcare services [46–48,51]. The articles discussed the importance of flagging social determinants of health-related barriers to care and providing additional support as needed to EDCs, including encouraging engagement with community resources [47,49,54,55,57].

***Instrumental supports provided by navigators*.** “Instrumental supports” refer to the tangible help provided to EDCs by navigators. Studies described navigators working with EDCs to prepare them for treatment appointments (e.g., providing education about mental health concerns and preparing what to discuss with their physician), create safety plans for suicide prevention, provide practical support to improve ease of resource engagement (e.g., help with insurance applications, documentation, scheduling appointments), and ensure services are received in a timely manner [46,51,57,58].

***Engaging circles of support to enable equitable care*.** In numerous studies, navigators played a crucial role in engaging EDCs’ circles of support who could help address daily needs or encourage service use [46,47,49,51,52,55,57,59]. This included involving family members in the navigation process [49,51], facilitating effective patient-clinician communication [55,57], ensuring clear communication between navigators and providers to deliver integrated care [47,52], promoting continuity of care to prevent readmission after discharge [46,49,51,59], and assessing formal and informal supports in EDCs’ lives [49].

***Psychological supports provided by navigators.*** Navigators also directly provided helpful psychological assistance, including delivering psychosocial and emotional support. For example, EDCs felt well supported when navigators offered positive encouragement, enhanced their self-esteem, presented psychoeducation, reinforced coping and problem-solving strategies, actively listened to needs and concerns, and provided counselling (e.g., to increase motivation toward treatment engagement) [49,51,52,58,60]. EDCs also appreciated feeling like they could be vulnerable with their navigator, especially when navigators had similar lived experiences that fostered a deeper sense of empathy and unique insight [46,51,52,58].

***Optimizing the patient experience.*** Furthermore, navigators worked to optimize EDCs’ experiences by empowering them to participate in their own care, encouraging their autonomy and sense of agency (e.g., by offering decision aids), involving them in decision-making processes, and building strong rapport to establish trust [48,49,51–53,55,57,58,61]. Studies found that empowering EDCs to voice their concerns with a safe, accessible, and nonjudgmental navigator can improve mental health symptoms and likelihood of remaining engaged/responsive in treatment [57, 61].

#### 3.2.4. Design and delivery of inclusive MHA navigation programs.

Operational practices, improvement models, frequency, delivery modes, and socially-inclusive practices need to be considered in navigation programming to appropriately support EDCs.

***Operational practices in navigation programs.*** Studies emphasized the need for standardized training and protocols for navigators [46, 47, 49, 51, 56]. This includes ensuring navigators are made aware of important basic values when supporting EDCs, such as being recovery-focused, empowering, and accepting [46]. In addition, comprehensive training on skills to effectively work with EDCs was noted as a critical component of program delivery, including reflective listening, motivational interviewing, using a strengths-based and harm reduction approach, facilitating interpersonal problem solving, and providing trauma-informed care [47, 49, 51]. Beyond direct patient interaction skills, EDC clients felt that navigators must be primed to manage their own roles (i.e., setting boundaries and managing burnout) and have a strong understanding of the healthcare system and suitable resources [46]. Navigators also expressed the importance of supervisors being available, supportive, and communicative [51]. Moreover, Koob et al. [47] identified the importance of standardizing protocols based on existing evidence to better address EDCs’ specific needs, improve individual outcomes, and allow for clearer, generalizable evaluations across navigation programs that can inform practice [47]. Furthermore, studies note the importance of establishing the location in which navigation is commenced (e.g., during inpatient care, prior to discharge), taking into consideration the target population’s needs and where groups may be best engaged [49,56].

Studies also highlighted outreach to areas that do not offer navigation services as a crucial strategy for engaging EDCs. For instance, Quintiliani et al. [56] noted that many safety-net hospitals do not offer screening or referrals for social needs influencing health. The authors suggest that offering navigation during patients’ stays in these hospitals may improve reach and efficiency [56]. Koob et al. [47] proposed that efficiency can be further built into navigation programs by embracing emerging technologies to manage cases (e.g., using an integrated database to streamline data collection, merge personal health and referral data, and reduce errors from manual data entry), thereby enhancing navigators’ capacity to support vulnerable groups [47].

***Equity-driven improvement models.*** In addition to integrating new technologies, studies report the need for equity-driven improvements to existing referral systems and processes to enhance support for EDCs [47,57]. Koob et al. [47] suggest establishment of closed loop referral systems to reduce miscommunication across the care team and improve fragmented care experiences. These systems can be used to clearly communicate referral information between primary care providers, specialists, and navigators, thereby providing better opportunities for follow-up [47]. Most importantly, van der Star et al. [58] stressed that it is crucial for programs to continuously seek and implement patient feedback to ensure EDCs’ needs are comprehensively addressed [58].

***Accessible navigation schedules.*** Across the included studies, EDCs’ desire for more flexible scheduling was evident. For instance, unstable work hours often made it difficult for individuals belonging to EDCs to attend sessions with their navigator [55]. Greater flexibility allowed navigators to meet individual, changing needs [51,57]. The length of navigation varied between different programs discussed in the studies, ranging from three months to six months [49,57,58,60,62]. Some studies expressed the need for a longer program length to circumvent EDCs’ ongoing barriers to care and to combat MHA disparities [49,56,58,61]. EDCs also commonly emphasized a desire for more frequent and/or more regular engagement with their navigator [49,51].

***Accessible navigation delivery modes.*** Many studies commented on accessible modes of delivery for navigation programs, including virtual navigation, in-person navigation, or a combination of contact methods. Virtual remote sessions (via phone or videoconference) were found to make program engagement easier for EDCs as they offer greater flexibility [55,58]. Despite being less frequently incorporated in navigation programs, text messaging was a delivery method that proved to be a popular form of communication among EDCs and may merit further exploration [56]. Some studies, however, noted that virtual delivery methods can be challenging for patients who do not have reliable access to a phone (mobile or landline) or other device. This may exacerbate inequities in service accessibility, such as among low-income groups or remote populations. An initial in-person visit may be beneficial to facilitate trust and develop stronger rapport for program engagement [55, 57]. Eliacin et al. [57] noted that participants recommended offering multiple delivery methods for navigation, such as telephone/videoconference alongside in-person visits to accommodate different preferences.

***Socially-inclusive navigation practices.*** Socially-inclusive practices to meet EDCs’ needs, such as personalizing support and encouraging use of community resources matched to individual goals, were also highlighted in the reviewed studies. Most studies implemented peer navigators who had shared identities with participants (i.e., race concordance, gender concordance, etc.). This was found to foster stronger relationships, where EDCs felt that their navigator could better relate to their concerns without judgment. Navigators who had overcome similar personal experiences were able to enhance motivation to engage in treatment among EDCs [51, 52, 54, 55, 57, 58, 61, 62]. Having lived experience was also described as helping to reduce stigma surrounding MHA and create a more welcoming care experience for EDCs overall [54]. In one example, Eliacin et al. [57] found that a lack of gender concordance may be a barrier to engagement with navigation, such as among patients who have experienced sexual trauma. Similarly, Quintiliani et al. [60] suggested that a lack of racial or ethnic concordance may have reduced receptiveness to patient navigation in their study. Furthermore, ensuring navigation is linguistically accessible and tailored to individual circumstances were also important considerations in the included studies, in recognition of the added social barriers EDCs face [46, 47, 49, 53, 55-62]. Tailoring to individual circumstances includes assessing individual stressors, risk, social supports, goals, and skills/strengths in order to offer the most suitable resources [49]. Studies also described the importance of navigators being well-equipped to provide culturally sensitive supports to remain respectful of EDCs and their individual values/expectations; this includes offering community resources matched to unique needs and diverse backgrounds [46, 47, 49, 51–54, 58, 62].

## 4. Discussion

This scoping review examined how MHA navigation programs support EDCs, including low-income individuals, racial and ethnic minorities, members of 2SLGBTQI+ communities, people with disabilities, Indigenous Peoples, and newcomers. The limited number of identified studies and recent publication dates of those included, highlights the emergence of MHA navigation programs over approximately the last decade, the need for additional research to inform practices, and the importance of equity-driven approaches to build inclusivity. Four key themes were identified from synthesis of 16 sources: barriers to equitable access to MHA services, influences on equitable service utilization, supports provided by MHA navigation to address complex needs, and design and delivery of inclusive MHA navigation programs.

Collectively, the themes identified in this review indicate the importance of a holistic, person-centered approach when helping EDCs navigate the MHA system. Navigators must consider MHA in the context of intersecting individual and systemic barriers to most appropriately address the needs of these historically marginalized populations, thereby improving engagement with care and client outcomes. Facilitators of MHA service use and engagement, such as via a strengths-based approach, can be leveraged to effectively provide tailored support and empower clients to overcome barriers to care [54, 58, 59, 61]. Other existing research echoes these sentiments, where clients’ positive perceptions of navigation have been associated with being offered resources/information matched to individual barriers and facilitators [63, 64].

Characteristics of navigators that promote engagement and relationship-building with clients belonging to EDCs were also emphasized through themes focused on promoting equitable service utilization and designing inclusive navigation programs. Navigators who have similar lived experiences and/or shared identities (gender, race, etc.) with EDCs are valuable, being able to uniquely relate to clients’ challenges, build strong rapport, and act as a source of motivation to engage in resources (e.g., clients feel inspired by peer navigators who have overcome MHA struggles) [46, 51, 54, 57]. Other literature has similarly shown that peer MHA navigation support can minimize the impacts of stigma, encourage treatment-seeking behaviors, improve recovery outcomes, and reduce hospital readmission rates [65–67]. Peer workers offer a special lens, being able to bridge gaps between patients and healthcare providers (i.e., restoring trust, facilitating communication, etc.) by using their prior experiences to develop creative, flexible solutions [68 - 70]. Adaptability is another important trait, with effective navigators being able to accommodate different concerns, embrace emerging technologies, and commit to cultural humility [46, 57, 61]. To offer culturally safe support, navigators should recognize that EDCs may have misconceptions about treatments or limited trust in service providers stemming from prior negative experiences in the healthcare system [46, 49, 54, 57]. Moreover, navigators who are non-judgmental, empathic, reliable, and are skillful in active listening are well suited to promote open conversations with EDCs [57, 61]. This further encourages EDCs to exercise their autonomy and participate in shared decision-making regarding their care [46, 71]. In addition to assessing these characteristics during hiring processes, MHA navigation programs should ensure ongoing training is provided to navigators [46]. EDCs can be better supported when navigators are educated on program values, strategies to self-regulate difficult emotions that may arise, and the importance of remaining respectful of varying perspectives (i.e., cultural competence) [46, 49, 69].

When considering features of equitable navigation programs, accessibility is identified as a top priority by studies both with and without a focus on MHA [51, 54, 72]. Examples of how navigation programs ensure accessibility include offering multiple delivery modes (e.g., phone calls, texting, e-mail, in-person visits), flexible scheduling, more frequent or longer-term navigation for clients with complex needs, and community outreach to improve awareness [55, 61]. Resources suggested to clients by navigators should also be easily accessible, considering the individual barriers they may face. For example, local or virtual resources may be more suitable for low-income clients who struggle to afford transportation services [51, 58, 59].

While the majority of sources in this review agreed on tailoring navigation support for EDCs, one included study on a peer-led MHA navigation program for minority Veterans had conflicting results [53]. Eliacin et al. [53] reported that participants in a pre-implementation evaluation felt that focusing on racial and ethnic identities and using a social determinants of health framework may be divisive, undermining social cohesion. Concerns around the need for additional supervision/training and difficulty of implementation were also emphasized. Moreover, some peer navigators argued that focusing on social determinants of health is redundant to their work, since all Veterans should be treated the same [53]. These findings highlight the importance of ensuring navigators’ values align with program goals and educating staff on healthcare disparities among minority groups. Other existing literature on navigation programs outside of the MHA system have similarly underscored the importance of offering enhanced cultural competency training to staff and maintaining a person-centered approach to effectively address care inequities [73, 74]. By shifting to an equity-centered approach, navigation programs can close perpetual gaps in patient outcomes and promote social inclusion of EDCs [75].

This scoping review synthesized promising navigation practices for supporting EDCs. While the discussed practices can be universally beneficial based on other literature on MHA navigation, they are not always consistently implemented together within individual programs [32]. This review points to the importance of recognizing the challenges uniquely faced by EDCs and intentionally incorporating as many of the identified practices/features as possible to make programs maximally accessible, responsive, and helpful for these groups. Overall, the findings underscore the importance of a deeper focus on equity and holistic support for marginalized populations in MHA navigation.

This scoping review involved a comprehensive multidisciplinary database search; however, there are important limitations to be considered. Only peer-reviewed studies written in English were included, potentially excluding other relevant articles and grey literature about supporting diverse communities. All included studies originated from the United States, which may have been due in part to these inclusion/exclusion criteria. While this may limit the transferability of findings to other settings, the identified themes may remain useful in supporting MHA navigation programs identify the most suitable strategies to adopt to better support EDCs. This review also did not segregate results by each EDC given that approaches largely overlapped across all included studies and individuals often identify with more than one social group. Assessing differences between specific equity-deserving groups, as well as in comparison with other dominant groups, may be a focus of future research. As this review prioritized qualitative data to comprehensively describe approaches used in MHA navigation, further research assessing quantitative evidence is warranted to offer additional insight into outcomes. Finally, studies focused on conditions outside of MHA were excluded. While this was meant to ensure relevance to the research question, literature on equitable navigation approaches in other areas of healthcare may contain valuable information that can be translated to the MHA system. Future work may explore approaches to supporting EDCs in other areas of healthcare.

## 5. Conclusion

Key considerations and strategies for MHA navigation programs when working with EDCs were identified in this scoping review. The importance of holistic, tailored care was evident from discussions around barriers to equitable access to MHA services, influences on equitable service utilization, supports provided by MHA navigation to address complex needs, and the design and delivery of inclusive MHA navigation programs. Also evident was the need for clear protocols, training, and ongoing support for navigators in order to ensure their consistent delivery of high-quality care. Integrating these themes is vital to ensuring EDCs are able to equitably access and benefit from navigation services, consistently feel heard and respected, and are offered resources well-suited to their needs. By examining critical considerations in providing navigation support to EDCs, this review offers key approaches for optimizing MHA system experiences and closing gaps in disparate outcomes for marginalized patients.

## Supporting information

S1 File PRISMA-ScR checklist. (DOCX)

S2 File Search strategies. (DOCX)

## References

[1] Castaldelli-Maia JM, Bhugra D. Analysis of global prevalence of mental and substance use disorders within countries: focus on sociodemographic characteristics and income levels. Int Rev Psychiatry. 2022;34(1):6–15. doi: 10.1080/09540261.2022.2040450 35584016

[2] Moitra M, Owens S, Hailemariam M, Wilson KS, Mensa-Kwao A, Gonese G, et al. Global Mental Health: Where We Are and Where We Are Going. Curr Psychiatry Rep. 2023;25(7):301–11. doi: 10.1007/s11920-023-01426-8 37256471 PMC10230139

[3] Vigo DV, Stein DJ, Harris MG, Kazdin AE, Viana MC, Munthali R, et al. Effective Treatment for Mental and Substance Use Disorders in 21 Countries. JAMA Psychiatry. 2025;82(4):347–57. doi: 10.1001/jamapsychiatry.2024.4378 39908011 PMC11800122

[4] Freeman HP, Rodriguez RL. History and principles of patient navigation. Cancer. 2011;117(15 Suppl):3539–42. doi: 10.1002/cncr.26262 21780088 PMC4557777

[5] Kourgiantakis T, Markoulakis R, Hussain A, Lee E, Ashcroft R, Williams C, et al. Navigating inequities in the delivery of youth mental health care during the COVID-19 pandemic: perspectives of youth, families, and service providers. Can J Public Health. 2022;113(6):806–16. doi: 10.17269/s41997-022-00670-4 35852728 PMC9663755

[6] Cénat JM, Kogan C, Noorishad P-G, Hajizadeh S, Dalexis RD, Ndengeyingoma A, et al. Prevalence and correlates of depression among Black individuals in Canada: The major role of everyday racial discrimination. Depress Anxiety. 2021;38(9):886–95. doi: 10.1002/da.23158 33949750

[7] Halladay J, Georgiades K, MacKillop J, Lipman E, Pires P, Duncan L. Identifying patterns of substance use and mental health concerns among adolescents in an outpatient mental health program using latent profile analysis. Eur Child Adolesc Psychiatry. 2024;33(3):739–47. doi: 10.1007/s00787-023-02188-7 36947251 PMC10031175

[8] Sobers M, Smith PM, Massaquoi N, Hamilton HA, Gesink D. Mental health service use among Black adolescents in Ontario by sex and distress level: a cross-sectional study. CMAJ. 2025;197(29):E901–14. doi: 10.1503/cmaj.241733 40921464 PMC12448776

[9] Government of Canada. Guide on Equity, Diversity and Inclusion Terminology. https://www.noslangues-ourlanguages.gc.ca/en/publications/equite-diversite-inclusion-equity-diversity-inclusion-eng 2025.

[10] Edalati H, Katan C, Taha S. Evaluation of the outcomes of equity-deserving individuals receiving services and support from integrated substance use health and mental health services: a pilot study protocol. Front Psychiatry. 2024;15:1425514. doi: 10.3389/fpsyt.2024.1425514 39720432 PMC11666509

[11] Braveman P. Health Inequalities, Disparities, Equity: What’s in a Name?. Am J Public Health. 2025;115(7):996–1002. doi: 10.2105/AJPH.2025.308062 40499108 PMC12160630

[12] Bresnahan M, Zhuang J. Culturally safe healthcare: changing the lens from provider control to patient agency. J Commun Healthc. 2024;17(3):244–53. doi: 10.1080/17538068.2024.2323856 38426444

[13] Power C, O’Neill K, Ng S-K, Berry E, Grigg M, Williams G, et al. “Because I couldn't understand and respond”: A mixed-method study examining the impact of language barriers on patient experiences of intensive care unit outreach team care. Australian Critical Care. 2025;38(3). 10.1016/j.aucc.2025.101198 39922097

[14] Webster CS, Taylor S, Thomas C, Weller JM. Social bias, discrimination and inequity in healthcare: mechanisms, implications and recommendations. BJA Educ. 2022;22(4):131–7. doi: 10.1016/j.bjae.2021.11.011 35531078 PMC9073302

[15] Farahi SMMM, Cénat JM. Racial disparities in the prevalence and determinants of anxiety symptoms among Arab, Asian, Black, Indigenous, White, and Mixed-racial individuals in Canada: The major role of racial discrimination. Psychiatry Research. 2025;353:116710. 40961912 10.1016/j.psychres.2025.116710

[16] Kingsbury M, Hammond NG, Johnstone F, Colman I. Suicidality among sexual minority and transgender adolescents: a nationally representative population-based study of youth in Canada. CMAJ. 2022;194(22):E767–74. doi: 10.1503/cmaj.212054 35667666 PMC9177208

[17] Adams LM, Miller AB. Mechanisms of Mental-Health Disparities Among Minoritized Groups: How Well Are the Top Journals in Clinical Psychology Representing This Work?. Clinical Psychological Science. 2021;10(3):387–416. doi: 10.1177/21677026211026979 35602543 PMC9122282

[18] Jenkins EK, McAuliffe C, Hirani S, Richardson C, Thomson KC, McGuinness L, et al. A portrait of the early and differential mental health impacts of the COVID-19 pandemic in Canada: Findings from the first wave of a nationally representative cross-sectional survey. Prev Med. 2021;145:106333. doi: 10.1016/j.ypmed.2020.106333 33509605 PMC9755654

[19] Lin SL. Inequities in Mental Health Care Facing Racialized Immigrant Older Adults With Mental Disorders Despite Universal Coverage: A Population-Based Study in Canada. J Gerontol B Psychol Sci Soc Sci. 2023;78(9):1555–71. doi: 10.1093/geronb/gbad036 36842070 PMC10461535

[20] Manning RB, Cipollina R, Lowe SR, Bogart KR, Ostrove JM, Adler JM, et al. Barriers to mental health service use among people with disabilities during the COVID-19 pandemic. Rehabil Psychol. 2023;68(4):351–61. doi: 10.1037/rep0000512 37470994 PMC10799191

[21] Sundareswaran M, Martignetti L, Purkey E. Barriers to primary care among immigrants and refugees in Peterborough, Ontario: a qualitative study of provider perspectives. BMC Prim Care. 2024;25(1):199. doi: 10.1186/s12875-024-02453-x 38840096 PMC11151623

[22] Forray AI, Oltean O, Hanft-Robert S, Madzamba R, Liem A, Schouten B. Uncovering multi-level mental healthcare barriers for migrants: A qualitative analysis across China, Germany, Netherlands, Romania, and South Africa. BMC Public Health. 2024;24. 10.1186/s12889-024-19046-z PMC11177472 38877460

[23] Olukotun M, Olanlesi-Aliu A, Idi Y, Ladha T, Bailey P, King R, et al. Institutional and systemic barriers and facilitators affecting healthcare access for Black women in Alberta. SSM - Qualitative Research in Health. 2024;6:100485. doi: 10.1016/j.ssmqr.2024.100485

[24] Salam Z, Carranza M, Newbold B, Wahoush O, Joseph A. Racialized Immigrants’ Encounters of Barriers and Facilitators in Seeking Mental Healthcare Services in Ontario, Canada. Community Ment Health J. 2025;61(3):556–67. doi: 10.1007/s10597-024-01362-8 39316360

[25] Ni Chaoilte A, Sanchez Clemente N, Lilygreen R, Ward A, Longley N, Eisen S. Exploring healthcare priorities, barriers, access and experiences of a family-centred approach among families seeking asylum in North London. BMJ Public Health. 2025;3(2):e001939. doi: 10.1136/bmjph-2024-001939 41133248 PMC12542707

[26] Nguyen NH, Subhan FB, Williams K, Chan CB. Barriers and Mitigating Strategies to Healthcare Access in Indigenous Communities of Canada: A Narrative Review. Healthcare (Basel). 2020;8(2):112. doi: 10.3390/healthcare8020112 32357396 PMC7349010

[27] Anthonisen G, Luke A, MacNeill L, MacNeill AL, Goudreau A, Doucet S. Patient navigation programs for people with dementia, their caregivers, and members of the care team: a scoping review. JBI Evid Synth. 2023;21(2):281–325. doi: 10.11124/JBIES-22-00024 36449660 PMC10578521

[28] Tremblay ES, Tartarilla AB, McCollister K, Umali M, Whitley MY, Ainsworth J, et al. A Pilot Study of the Feasibility and Acceptability of a Patient Navigator in a Pediatric Type 1 Diabetes Clinic. Can J Diabetes. 2025;49(7):355-364.e2. doi: 10.1016/j.jcjd.2025.05.011 40505961 PMC13014301

[29] Salabarría-Peña Y, Douglas C, Brantley M, Johnson AK. Informing the future of PrEP navigation: Findings from a five-site cluster evaluation. Eval Program Plann. 2022;90:101999. doi: 10.1016/j.evalprogplan.2021.101999 34503854 PMC11288482

[30] Markoulakis R, Cader H, Wong K, Kodeeswaran S, Addison T, Walsh C, et al. The role of navigation services in supporting mental health and addictions care transitions: A qualitative exploration of perspectives from transitional-aged youth, family, and service providers (part 2). Health Care Transit. 2024;3:100082. doi: 10.1016/j.hctj.2024.100082 39712476 PMC11657732

[31] Markoulakis R, Luke A, Reid A, Mehra K, Levitt A, Doucet S. Proceedings of the inaugural Canadian Healthcare Navigation Conference: a forum for sharing innovations and best practices in navigation services. BMC Proc. 2021;15(Suppl 16):24. doi: 10.1186/s12919-021-00229-0 34844595 PMC8629593

[32] Mullen JN, Levitt A, Markoulakis R. Supporting Individuals with Mental Health and/or Addictions Issues Through Patient Navigation: A Scoping Review. Community Ment Health J. 2023;59(1):35–56. doi: 10.1007/s10597-022-00982-2 35648257

[33] Waid J, Halpin K, Donaldson R. Mental health service navigation: a scoping review of programmatic features and research evidence. Social Work in Mental Health. 2021;19(1):60–79. doi: 10.1080/15332985.2020.1870646

[34] Kokorelias KM, Shiers-Hanley JE, Rios J, Knoepfli A, Hitzig SL. Factors Influencing the Implementation of Patient Navigation Programs for Adults with Complex Needs: A Scoping Review of the Literature. Health Serv Insights. 2021;14:11786329211033267. doi: 10.1177/11786329211033267 34349519 PMC8287353

[35] Chan RJ, Milch VE, Crawford-Williams F, Agbejule OA, Joseph R, Johal J, et al. Patient navigation across the cancer care continuum: An overview of systematic reviews and emerging literature. CA Cancer J Clin. 2023;73(6):565–89. doi: 10.3322/caac.21788 37358040

[36] Haroen H, Agustina HR, Pahria T, Mambang Sari CW, Adhipurnawan Winarno GN, Bangun AV, et al. A Scoping Review of Patient Navigation in the Continuity of Cancer Care for Women. Int J Womens Health. 2025;17:4779–98. doi: 10.2147/IJWH.S558219 41322375 PMC12664311

[37] Arksey H, O’Malley L. Scoping studies: towards a methodological framework. International Journal of Social Research Methodology. 2005;8(1):19–32. doi: 10.1080/1364557032000119616

[38] Peters MDJ, Marnie C, Tricco AC, Pollock D, Munn Z, Alexander L, et al. Updated methodological guidance for the conduct of scoping reviews. JBI Evid Synth. 2020;18(10):2119–26. doi: 10.11124/JBIES-20-00167 33038124

[39] Tricco AC, Lillie E, Zarin W, O’Brien KK, Colquhoun H, Levac D, et al. PRISMA Extension for Scoping Reviews (PRISMA-ScR): Checklist and Explanation. Ann Intern Med. 2018;169(7):467–73. doi: 10.7326/M18-0850 30178033

[40] VonVille H. Ovid Medline Search Filters: Economic Stability: Poverty, Social Class, etc. https://hsls.libguides.com/Ovid-Medline-search-filters

[41] Wafford QE, Miller CH, Wescott AB, Kubilius RK. Meeting a need: development and validation of PubMed search filters for immigrant populations. J Med Libr Assoc. 2024;112(1):22–32. doi: 10.5195/jmla.2024.1716 38911528 PMC11189137

[42] VonVille H. Ovid Medline Search Filters: Sexual minorities: University of Pittsburgh Health Science Library System Guides; 2025 https://hsls.libguides.com/Ovid-Medline-search-filters

[43] Harding L, DeCaire R, Ellis U, Delaurier-Lyle K, Schillo J, Turin M. Language improves health and wellbeing in Indigenous communities: A scoping review. Language and Health. 2025;3(1):100047. doi: 10.1016/j.laheal.2025.100047

[44] Ioerger M, Flanders RM, Goss KD, Turk MA. Developing a systematic search strategy related to people with disability: A brief report testing the utility of proposed disability search terms in a search about opioid use. Disabil Health J. 2019;12(2):318–22. doi: 10.1016/j.dhjo.2018.11.009 30470478

[45] MAXQDA. All-in-One Qualitative & Mixed Methods Data Analysis Tool. https://www.maxqda.com/ 2026.

[46] Corrigan PW, Torres A, Lara JL, Sheehan L, Larson JE. The Healthcare Needs of Latinos with Serious Mental Illness and the Potential of Peer Navigators. Adm Policy Ment Health. 2017;44(4):547–57. doi: 10.1007/s10488-016-0737-2 27236458 PMC5997453

[47] Koob C, Stuenkel M, Gagnon RJ, Griffin SF, Sease K. Examining Patient- and Community-Level Factors Associated with Pediatric Mental Healthcare Access Within a Patient Navigation Program. Community Ment Health J. 2024;60(6):1055–67. doi: 10.1007/s10597-024-01258-7 38507129 PMC11199227

[48] Silverstein M, Diaz-Linhart Y, Grote N, Cadena L, Cabral H, Feinberg E. Harnessing the Capacity of Head Start to Engage Mothers with Depression in Treatment. J Health Care Poor Underserved. 2017;28(1):14–23. doi: 10.1353/hpu.2017.0003 28238982

[49] Vance MM, Gryglewicz K, Nam E, Richardson S, Borntrager L, Karver MS. Exploring Service Use Disparities among Suicidal Black Youth in a Suicide Prevention Care Coordination Intervention. J Racial Ethn Health Disparities. 2023;10(5):2231–43. doi: 10.1007/s40615-022-01402-7 36100810

[50] Government of Canada. Best Practices in Equity, Diversity and Inclusion in Research Practice and Design. 2025. https://sshrc-crsh.canada.ca/funding-financement/nfrf-fnfr/edi-eng.aspx

[51] Sheehan L, Torres A, Lara JL, Paniagua D, Larson JE, Mayes J, et al. Qualitative Evaluation of a Peer Navigator Program for Latinos with Serious Mental Illness. Adm Policy Ment Health. 2018;45(3):495–504. doi: 10.1007/s10488-017-0839-5 29168016 PMC5997452

[52] Corrigan PW, Pickett S, Batia K, Michaels PJ. Peer navigators and integrated care to address ethnic health disparities of people with serious mental illness. Soc Work Public Health. 2014;29(6):581–93. doi: 10.1080/19371918.2014.893854 25144699 PMC5371355

[53] Eliacin J, Matthias MS, Burgess DJ, Patterson S, Damush T, Pratt-Chapman M, et al. Pre-implementation Evaluation of PARTNER-MH: A Mental Healthcare Disparity Intervention for Minority Veterans in the VHA. Adm Policy Ment Health. 2021;48(1):46–60. doi: 10.1007/s10488-020-01048-9 32399857 PMC9645234

[54] Giraldo A, Shah P, Zerbo E, Nyaku AN. The role of recovery peer navigators in retention in outpatient buprenorphine treatment: a retrospective cohort study. Ann Med. 2024;56(1):2355566. doi: 10.1080/07853890.2024.2355566 38823420 PMC11146239

[55] Eliacin J, Burgess D, Rollins AL, Patterson S, Damush T, Bair MJ, et al. Outcomes of a peer-led navigation program, PARTNER-MH, for racially minoritized Veterans receiving mental health services: a pilot randomized controlled trial to assess feasibility and acceptability. Transl Behav Med. 2023;13(9):710–21. doi: 10.1093/tbm/ibad027 37130337

[56] Quintiliani LM, Kathuria H, Truong V, Murillo J, Borrelli B, Xuan Z, et al. Patient navigation among recently hospitalized smokers to promote tobacco treatment: Results from a randomized exploratory pilot study. Addict Behav. 2021;113:106659. doi: 10.1016/j.addbeh.2020.106659 33010473 PMC7946867

[57] Eliacin J, Matthias MS, Cameron KA, Burgess DJ. Veterans’ views of PARTNER-MH, a peer-led patient navigation intervention, to improve patient engagement in care and patient-clinician communication: A qualitative study. Patient Educ Couns. 2023;114:107847. doi: 10.1016/j.pec.2023.107847 37331280 PMC11184508

[58] van der Star A, Randall A, Salgin L, Brady JP, Albright C, Mitzner J, et al. Development of a Suicide Prevention Intervention for Sexual and Gender Minority Youth and Young Adults: Rationale, Design, and Evidence of Feasibility and Acceptability. Suicide Life Threat Behav. 2025;55(2):e70014. doi: 10.1111/sltb.70014 40179218 PMC11968012

[59] Running Bear U, Poole EM, Muller C, Hanson JD, Noonan C, Trojan J, et al. The use of patient navigation to transition detoxification patients to substance use treatment in the Alaska Interior. Public Health Pract (Oxf). 2023;6:100418. doi: 10.1016/j.puhip.2023.100418 37635913 PMC10448195

[60] Quintiliani LM, Truong V, Ulrich ME, Murillo J, Jean C, Xuan Z, et al. Process evaluation of counseling delivered by a patient navigator in an efficacious smoking cessation intervention among low-income primary care patients. Addict Behav Rep. 2019;9:100176. doi: 10.1016/j.abrep.2019.100176 31193812 PMC6542736

[61] Poleshuck EL, Juskiewicz I, Wittink M, Crean H, Cerulli C. Is a patient navigation program more helpful than a referral program for reducing depression and improving quality of life among women living in neighborhoods with few resources?. 2019. 38232195

[62] Lasser KE, Quintiliani LM, Truong V, Xuan Z, Murillo J, Jean C, et al. Effect of Patient Navigation and Financial Incentives on Smoking Cessation Among Primary Care Patients at an Urban Safety-Net Hospital: A Randomized Clinical Trial. JAMA Intern Med. 2017;177(12):1798–807. doi: 10.1001/jamainternmed.2017.4372 29084312 PMC5820724

[63] Rattray M, Shelby-James T. Clients’ experiences of receiving service navigation for mental health support in primary care: findings from a mixed-methods evaluation. BMC Health Serv Res. 2025;25(1):445. doi: 10.1186/s12913-025-12622-y 40148880 PMC11948767

[64] Noriega Esquives BS, Moreno PI, Munoz E, Lad TE, Hollowell CMP, Benzo RM, et al. Effects of a culturally tailored patient navigation program on unmet supportive care needs in Hispanic/Latino cancer survivors: A randomized controlled trial. Cancer. 2025;131(1):e35626. doi: 10.1002/cncr.35626 39487386 PMC11694333

[65] Hailemariam M, Weinstock LM, Sneed RS, Taylor B, Corrigan PW, Johnson JE. Peer navigation intervention for individuals with serious mental illness reentering the community after jail incarceration: a qualitative case study. Pilot Feasibility Stud. 2024;10(1):129. doi: 10.1186/s40814-024-01555-8 39438944 PMC11494813

[66] Peart A, Horn F, Rowland B, Arunogiri S, Jones D, Manning V, et al. Peer navigation: a pilot study to improve recovery capital for alcohol and other drug telephone helpline callers. Drugs: Education, Prevention and Policy. 2024;32(2):164–74. doi: 10.1080/09687637.2024.2330941

[67] Magidson JF, Regan S, Powell E, Jack HE, Herman GE, Zaro C, et al. Peer recovery coaches in general medical settings: Changes in utilization, treatment engagement, and opioid use. J Subst Abuse Treat. 2021;122:108248. doi: 10.1016/j.jsat.2020.108248 33509420

[68] Lennox R, Lamarche L, O’Shea T. Peer support workers as a bridge: a qualitative study exploring the role of peer support workers in the care of people who use drugs during and after hospitalization. Harm Reduct J. 2021;18(1):19. doi: 10.1186/s12954-021-00467-7 33593364 PMC7885412

[69] Aghabayli F, Nielssen I, Zapata-Cardona LA, Ahmed S, Ezemenahi C, Parmar S, et al. From experience to a learning health system: peer-to-peer perspectives and implications for healthcare navigation in Alberta, Canada. Front Health Serv. 2025;5:1642188. doi: 10.3389/frhs.2025.1642188 41179850 PMC12575376

[70] Markoulakis R, Bowles K, Chan S, Weingust S, Dobbin K, Levitt A. Changes in Perception of Caregiving Experience Following Caregiver Peer Support Within a Mental Health and Addictions Navigation Service. Community Ment Health J. 2022;58(4):740–8. doi: 10.1007/s10597-021-00879-6 34365586

[71] Teggart K, Neil-Sztramko SE, Nadarajah A, Wang A, Moore C, Carter N, et al. Effectiveness of system navigation programs linking primary care with community-based health and social services: a systematic review. BMC Health Serv Res. 2023;23(1):450. doi: 10.1186/s12913-023-09424-5 37158878 PMC10165767

[72] Saluja K, Mahbub A, Gauthier AP, Lemonde M, Timony P, Durand F, et al. Understanding patient barriers and enablers to accessing community resources: a qualitative study to inform navigation service delivery. BMC Prim Care. 2025;26(1):322. doi: 10.1186/s12875-025-03029-z 41136927 PMC12551251

[73] Phillips S, Villalobos AVK, Crawbuck GSN, Pratt-Chapman ML. In their own words: patient navigator roles in culturally sensitive cancer care. Support Care Cancer. 2019;27(5):1655–62. doi: 10.1007/s00520-018-4407-7 30109486 PMC6449285

[74] Rankin A, Baumann A, Downey B, Valaitis R, Montour A, Mandy P. The Role of the Indigenous Patient Navigator: A Scoping Review. Can J Nurs Res. 2022;54(2):199–210. doi: 10.1177/08445621211066765 35014886 PMC9109580

[75] Waid J, Tomfohrde O, Kutzler C. Promoting health and social equity through family navigation to prevention and early intervention services: a proof of concept study. BMC Public Health. 2022;22(1):1972. doi: 10.1186/s12889-022-14320-4 36303175 PMC9610316

