# Supplementary material for: Pathways to inclusion: A scoping review exploring equity, diversity, and inclusion in mental health and addictions patient navigation programs
Source: PLOS Ment Health. 2026 Jun 25;3(6):e0000557. doi: 10.1371/journal.pmen.0000557 (PMC13298921; doi:10.1371/journal.pmen.0000557)
Supplement: S2 File — (DOCX) [file pmen.0000557.s002.docx]

# Ovid Medline and PsycInfo (Searches were identical)

1 "Sexual and Gender Minorities"/ 15230

2 lesbian*.mp. 10811

3 gay.mp. 17279

4 Queer.mp. 3931

5 homosexuality/ 12535

6 homosexual*.mp. 43774

7 homosexuality, female/ 4490

8 homosexuality, male/ 22713

9 MSM.mp. 15755

10 males who have sex with males.mp. 47

11 men who have sex with men.mp. 17906

12 "females who have sex with females".mp. 0

13 women who have sex with women.mp. 227

14 nonheterosex*.mp. 237

15 non-heterosex*.mp. 582

16 polysexual*.mp. 8

17 polyamor*.mp. 330

18 bisexuality/ 5232

19 bisexual*.mp. 16210

20 pansex*.mp. 263

21 "Transgender Persons"/ 9258

22 "gender identity"/ 23642

23 "Health Services for Transgender Persons"/ 224

24 transsexualism/ 4816

25 transgender*.mp. 18406

26 transmasc*.mp. 540

27 transfem*.mp. 8804

28 transm#n.mp. 499

29 trans m#n.mp. 453

30 transwom#n.mp. 393

31 trans wom#n.mp. 728

32 ((gender or sex) adj2 (change* or confirmation or disorder or divers* or dysphoria or identity or minorit* or nonconforming or non-conforming or reassignment or surgeries or surgery)).mp. 59071

33 genderqueer.mp. 226

34 non-binary.mp. 1734

35 nonbinary.mp. 1776

36 genderfluid.mp. 27

37 asexual*.mp. 12955

38 ace-spectrum.mp. 4

39 "Intersex Persons"/ 13

40 intersex.mp. 2635

41 LGB*.mp. 10323

42 GLB*.mp. 1388

43 2SLGB*.mp. 131

44 two spirit.mp. 231

45 1 or 2 or 3 or 4 or 5 or 6 or 7 or 8 or 9 or 10 or 11 or 12 or 13 or 14 or 15 or 16 or 17 or 18 or 19 or 20 or 21 or 22 or 23 or 24 or 25 or 26 or 27 or 28 or 29 or 30 or 31 or 32 or 33 or 34 or 35 or 36 or 37 or 38 or 39 or 40 or 41 or 42 or 43 or 44 141309

46 Poverty/ 46504

47 poverty*.mp. 80141

48 "social class"/ 47111

49 "socioeconomic factors"/ 182693

50 (((economic or economical or economically) adj2 (distress or distressed or factors)) or ((economically or socially or socioeconomically or stability or stable) adj1 disadvantaged) or income or poverty or ((social or socially or socioeconomic) adj2 (class or determinants or disparities or disparity or inequities or inequity or status))).mp. 427727

51 46 or 47 or 48 or 49 or 50 542847

52 "Ethnicity"/ 79011

53 (((children or communit* or faculty or men or patien* or people or person or persons or women) adj2 colo?r) or ethnic or ethnical or ethnically or ethnicized or ethnicities or ethnicism or ethnicities or ethnicity or ethnics or ethnic or minorities or minority or race or races or racial or racially).mp. 494734

54 Minority Groups/ 19554

55 racialized.mp. 1979

56 "Race Factors"/ 1428

57 "Racial Groups"/ 28413

58 Racism/ 8571

59 "Black or African American"/ 79288

60 ((African adj1 (American* Canadian* or ancestry)) or (Black adj1 (m#n or people or person* or student* or wom#n)) or Blacks).mp. 73624

61 "Hispanic or Latino"/ 37943

62 Hispanic.mp. 91782

63 latina*.mp. 6454

64 latino*.mp. 49441

65 latine*.mp. 703

66 latinx*.mp. 5041

67 52 or 53 or 54 or 55 or 56 or 57 or 58 or 59 or 60 or 61 or 62 or 63 or 64 or 65 or 66 603737

68 Indigenous Peoples/ 1862

69 Health Services, Indigenous/ 4698

70 Indigenous Canadians/ 326

71 (aborig* or indigen* or first nation* or first-nation* or first peoples or native people* or (alaska* adj3 native*) or inuit* or inu?k or Eskimo* or metis or (native* adj3 america*) or (hawai* adj2 native*) or pacific islander* or (pacific island* adj3 native*) or america* indian* or amerindian* or Torres Strait Island* or Maori*).mp. 110827

72 68 or 69 or 70 or 71 110827

73 Disabled Persons/ 49787

74 disab*.mp. 469663

75 Amputees/ 4823

76 Amputee*.mp. 8990

77 paraplegia/ 13366

78 parapleg*.mp. 25381

79 quadriplegia/ 8584

80 quadripleg*.mp. 11391

81 Persons with Hearing Impairments/ 3295

82 Hearing loss/ 22617

83 hearing loss.mp. 89710

84 deaf*.mp. 56954

85 visually impaired persons/ 2904

86 blindness/ 22202

87 (blind* not (double blind or single blind or blind study)).mp. 187743

88 Vision disorders/ 32214

89 developmental disabilities/ 23983

90 dependent ambulation/ 207

91 mobility limitation/ 5692

92 wheelchairs/ 5907

93 wheelchair.mp. 9032

94 Self-Help Devices/ 6388

95 Spinal cord injuries/ 48300

96 spinal cord injur*.mp. 66266

97 cerebral palsy/ 25628

98 cerebral palsy.mp. 35919

99 spinal dysraphism/ 7024

100 spina bifida.mp. 10541

101 multiple sclerosis/ 68792

102 multiple sclerosis.mp. 111879

103 Amyotrophic Lateral Sclerosis/ 26243

104 Amyotrophic Lateral Sclerosis.mp. 39448

105 als.mp. 82266

106 Brain injuries, traumatic/ 17220

107 traumatic brain injury.mp. 56848

108 muscular distrophies/ 0

109 muscualar dystrophy.mp. 2

110 Attention Deficit Disorder with Hyperactivity/ 38423

111 adhd.mp. 38345

112 attention deficit disorder.mp. 39416

113 Muscular Atrophy, Spinal/ 5591

114 spinal muscular atrophy.mp. 7892

115 muscular diseases/ 27598

116 myopathy.mp. 27792

117 Autism Spectrum Disorder/ 25438

118 ASD.mp. 43562

119 autism.mp. 77661

120 Down Syndrome/ 27696

121 down syndrome.mp. 33833

122 peripheral nervous system diseases/ 25904

123 peripheral neuropathy.mp. 26545

124 ((vision or visual* or hearing or mental or psychiatric or function* or activity or mobility or cognitive or intellectual) adj2 (limitation or limited or impair* or disab*)).mp. 423702

125 "Emigrants and Immigrants"/ or "Emigration and Immigration"/ or "Refugees"/ or "Transients and Migrants"/ or "Undocumented Immigrants"/ or "adopted country".mp. or alien.mp. or aliens.mp. or asylee.mp. or asylees.mp. or asylum.mp. or "au pairs".mp. or "au pair".mp. or "birth abroad".mp. or "birth countries".mp. or "birth country".mp. or "birth overseas".mp. or "born abroad".mp. or "born overseas".mp. or "border communities".mp. or "border community".mp. or "border health".mp. or "civil surgeon".mp. or "civil surgeons".mp. or citizenship.mp. or "countries of birth".mp. or "country of birth".mp. or "country of destination".mp. or "countries of origin".mp. or "country of origin".mp. or "customs and border protection".mp. or "deferred action for childhood arrivals".mp. or daca.mp. or "department of homeland security".mp. or deport.mp. or deportation.mp. or deportations.mp. or deported.mp. or "destination countries".mp. or "destination country".mp. or "diasporic communities".mp. or "diasporic community".mp. or "displaced person".mp. or "displaced persons".mp. or "displaced children".mp. or "displaced families".mp. or "displaced individuals".mp. or "displaced men".mp. or "displaced patients".mp. or "displaced people".mp. or "displaced peoples".mp. or "displaced women".mp. or "internationally displaced".mp. or "duration of residence".mp. or "ellis island".mp. or emigrate.mp. or emigrated.mp. or emigration.mp. or emigrant.mp. or emigrants.mp. or emigrating.mp. or "exchange student".mp. or "exchange students".mp. or "exchange visitor".mp. or "exchange visitors".mp. or exile.mp. or exiled.mp. or exiles.mp. or expatriation.mp. or expatriate.mp. or expatriates.mp. or expatriated.mp. or "family reunification".mp. or "family reunifications".mp. or "first generation".mp. or "first generations".mp. or "2.5 generation".mp. or "2.5 generations".mp. or "second generations".mp. or "third generation".mp. or "third generations".mp. or "mixed generation".mp. or "foreign birth".mp. or foreign-born.mp. or "foreign brides".mp. or "foreign citizen".mp. or "foreign citizens".mp. or "foreign citizenship".mp. or "foreign families".mp. or "foreign family".mp. or "foreign children".mp. or "foreign men".mp. or "foreign minor".mp. or "foreign minors".mp. or "foreign female".mp. or "foreign females".mp. or "foreign individuals".mp. or "foreign male".mp. or "foreign males".mp. or "foreign patient".mp. or "foreign patients".mp. or "foreign people".mp. or "foreign person".mp. or "foreign persons".mp. or "foreign resident".mp. or "foreign residents".mp. or "foreign national".mp. or "foreign nationals".mp. or "foreign nationalities".mp. or "foreign nationality".mp. or "foreign population".mp. or "foreign populations".mp. or "foreign woman".mp. or "foreign women".mp. or "foreigner".mp. or "foreigners".mp. or "green card".mp. or "green cards".mp. or "guest worker".mp. or "guest workers".mp. or "h 1b".mp. or "host countries".mp. or "host country".mp. or "host societies".mp. or "host society".mp. or immigrant.mp. or immigrants.mp. or immigrate.mp. or immigrated.mp. or immigrates.mp. or immigrating.mp. or immigration.mp. or "intercountry adoption".mp. or "intercountry adoptions".mp. or "international adoption".mp. or "international adoptions".mp. or "international student".mp. or "international students".mp. or "length of residence".mp. or "length of residency".mp. or "maternal nativity".mp. or migrant.mp. or migrants.mp. or migration.mp. or "migrated children".mp. or nativity.mp. or naturalization.mp. or naturalized.mp. or "new countries".mp. or "new country".mp. or "new entrant".mp. or "new entrants".mp. or "new settler".mp. or "new settlers".mp. or "non citizen".mp. or "non citizens".mp. or noncitizen.mp. or noncitizens.mp. or "non national".mp. or "non nationals".mp. or nonnational.mp. or nonnationals.mp. or nonnative.mp. or nonnatives.mp. or "non native".mp. or "non natives".mp. or "non refoulement".mp. or nonrefugee.mp. or nonrefugees.mp. or "overseas birth".mp. or "overseas born".mp. or "permanent residence".mp. or "permanent residency".mp. or "permanent resident".mp. or "permanent residents".mp. or "port health".mp. or postmigration.mp. or premigration.mp. or "receiving countries".mp. or "receiving country".mp. or "receiving societies".mp. or "receiving society".mp. or refugee.mp. or refugees.mp. or "region of origin".mp. or "regions of origin".mp. or "resettlement countries".mp. or "resettlement country".mp. or "seasonal farmworkers".mp. or "seasonal farm worker".mp. or "seasonal farm workers".mp. or "seasonal farmworker".mp. or "seasonal worker".mp. or "seasonal workers".mp. or "stateless people".mp. or "stateless person".mp. or "stateless persons".mp. or "stateless children".mp. or "stateless individuals".mp. or "stateless patients".mp. or "temporary protected status".mp. or "transnational community".mp. or "unaccompanied child".mp. or "unaccompanied children".mp. or "unaccompanied minor".mp. or "unaccompanied minors".mp. or undocumented.mp. or UNHCR.mp. or visa.mp. or visas.mp. or "voluntary return".mp. or xenophobia.mp. or xenophobic.mp. 582968

126 73 or 74 or 75 or 76 or 77 or 78 or 79 or 80 or 81 or 82 or 83 or 84 or 85 or 86 or 87 or 88 or 89 or 90 or 91 or 92 or 93 or 94 or 95 or 96 or 97 or 98 or 99 or 100 or 101 or 102 or 103 or 104 or 105 or 106 or 107 or 108 or 109 or 110 or 111 or 112 or 113 or 114 or 115 or 116 or 117 or 118 or 119 or 120 or 121 or 122 or 123 or 124 1629904

127 45 or 51 or 67 or 72 or 125 or 126 3302666

128 Mental Health/ 75947

129 mental health.mp. 342068

130 Mental Disorders/ 189792

131 Mental disorder*.mp. 243721

132 Mentally ill persons/ 6488

133 mental* illness*.mp. 47752

134 Anxiety/ 126022

135 anxiety.mp. 374430

136 Generalized Anxiety Disorder/ 166

137 Anxiety Disorders/ 45492

138 Depression/ 176341

139 Depressive Disorder, Major/ 43965

140 Depressive Disorder/ 76476

141 depression.mp. 572292

142 Suicide/ 43631

143 suicid*.mp. 131078

144 Suicidal Ideation/ 16599

145 Stress Disorders, Post-Traumatic/ 47001

146 PTSD.mp. 40703

147 post-traumatic stress.mp. 24192

148 cptsd.mp. 570

149 Alzheimer Disease/ 137797

150 Alzheimers.mp. 204344

151 Dementia/ 69537

152 Dementia.mp. 188191

153 Bipolar Disorder/ 48152

154 Bipolar.mp. 100644

155 bpd.mp. 14781

156 borderline personality.mp. 11930

157 Dissociative Identity Disorder/ 882

158 dissociative identity.mp. 1208

159 Schizophrenia/ 117259

160 Schizophrenia.mp. 174304

161 Schizophrenia, Disorganized/ 544

162 Altered Perception Syndrome.mp. 2

163 Schizophrenia, Paranoid/ 4260

164 Psychosis Spectrum Syndrome.mp. 1

165 Neuro-Emotional Integration Disorder.mp. 3

166 Schizophrenia, Catatonic/ 593

167 Schizophrenia, Childhood/ 1576

168 Schizophrenia, Treatment-Resistant/ 332

169 Addiction Medicine/ 305

170 addiction.mp. 70066

171 gambl*.mp. 14152

172 "substance use".mp. 64320

173 substance abuse.mp. 62212

174 alcoholism.mp. 93696

175 alcoholic.mp. 119660

176 alcohol dependence.mp. 11538

177 cocaine.mp. 49628

178 cigarette*.mp. 93964

179 heroin.mp. 22086

180 128 or 129 or 130 or 131 or 132 or 133 or 134 or 135 or 136 or 137 or 138 or 139 or 140 or 141 or 142 or 143 or 144 or 145 or 146 or 147 or 148 or 149 or 150 or 151 or 152 or 153 or 154 or 155 or 156 or 157 or 158 or 159 or 160 or 161 or 162 or 163 or 164 or 165 or 166 or 167 or 168 or 169 or 170 or 171 or 171 or 172 or 173 or 173 or 174 or 175 or 176 or 177 or 178 or 179 2213129

181 Patient navigation/ 1399

182 patient navigation.mp. 2317

183 navigation services.mp. 355

184 patient coordination.mp. 43

185 care coordination.mp. 7271

186 patient support services.mp. 50

187 181 or 182 or 183 or 184 or 185 or 186 9656

188 127 and 180 and 187 571

# Ovid EMBASE

Ovid MEDLINE(R) ALL <1946 to December 03, 2025>

1 "Sexual and Gender Minorities"/ 15230

2 lesbian*.mp. 10811

3 gay.mp. 17279

4 Queer.mp. 3931

5 bisexuality/ or homosexuality/ or "sexual and gender minority"/ or homosexual female/ or gender identity/ 39023

6 homosexual*.mp. 43774

7 MSM.mp. 15755

8 males who have sex with males.mp. 47

9 men who have sex with men.mp. 17906

10 "females who have sex with females".mp. 0

11 women who have sex with women.mp. 227

12 nonheterosex*.mp. 237

13 non-heterosex*.mp. 582

14 polysexual*.mp. 8

15 polyamor*.mp. 330

16 bisexual*.mp. 16210

17 pansex*.mp. 263

18 male to female transgender/ or female to male transgender/ or "transgender and gender nonbinary"/ or transgender/ 9258

19 transgender*.mp. 18406

20 transmasc*.mp. 540

21 transfem*.mp. 8804

22 transm#n.mp. 499

23 trans m#n.mp. 453

24 transwom#n.mp. 393

25 trans wom#n.mp. 728

26 ((gender or sex) adj2 (change* or confirmation or disorder or divers* or dysphoria or identity or minorit* or nonconforming or non-conforming or reassignment or surgeries or surgery)).mp. 59071

27 genderqueer.mp. 226

28 non-binary.mp. 1734

29 nonbinary.mp. 1776

30 genderfluid.mp. 27

31 asexual people/ 0

32 asexual*.mp. 12955

33 ace-spectrum.mp. 4

34 hermaphroditism/ or intersex/ 9764

35 intersex.mp. 2635

36 LGB*.mp. 10323

37 GLB*.mp. 1388

38 2SLGB*.mp. 131

39 two spirit.mp. 231

40 1 or 2 or 3 or 4 or 5 or 6 or 7 or 8 or 9 or 10 or 11 or 12 or 13 or 14 or 15 or 16 or 17 or 18 or 19 or 20 or 21 or 22 or 23 or 24 or 25 or 26 or 27 or 28 or 29 or 30 or 31 or 32 or 33 or 34 or 35 or 36 or 37 or 38 or 39 147948

41 extreme poverty/ or child poverty/ or poverty level/ or poverty/ or social class/ or lowest income group/ 90606

42 poverty*.mp. 80141

43 (((economic or economical or economically) adj2 (distress or distressed or factors)) or ((economically or socially or socioeconomically or stability or stable) adj1 disadvantaged) or income or poverty or ((social or socially or socioeconomic) adj2 (class or determinants or disparities or disparity or inequities or inequity or status))).mp. 427727

44 41 or 42 or 43 427735

45 ethnic group/ or ethnicity/ or race difference/ or African American/ or structural racism/ or vicarious racism/ or racism/ or scientific racism/ or medical racism/ or Hispanic/ 157953

46 (((children or communit* or faculty or men or patien* or people or person or persons or women) adj2 colo?r) or ethnic or ethnical or ethnically or ethnicized or ethnicities or ethnicism or ethnicities or ethnicity or ethnics or ethnic or minorities or minority or race or races or racial or racially).mp. 494734

47 racialized.mp. 1979

48 ((African adj1 (American* Canadian* or ancestry)) or (Black adj1 (m#n or people or person* or student* or wom#n)) or Blacks).mp. 73624

49 Hispanic.mp. 91782

50 latina*.mp. 6454

51 latino*.mp. 49441

52 latine*.mp. 703

53 latinx*.mp. 5041

54 45 or 46 or 47 or 48 or 49 or 50 or 51 or 52 or 53 603825

55 indigenous health care/ or Indigenous Australian/ or indigenous people/ or American Indian/ 7152

56 (aborig* or indigen* or first nation* or first-nation* or first peoples or native people* or (alaska* adj3 native*) or inuit* or inu?k or Eskimo* or metis or (native* adj3 america*) or (hawai* adj2 native*) or pacific islander* or (pacific island* adj3 native*) or america* indian* or amerindian* or Torres Strait Island* or Maori*).mp. 110827

57 55 or 56 110827

58 disabled person/ 51362

59 disab*.mp. 469663

60 amputee/ 4823

61 Amputee*.mp. 8990

62 spastic paraplegia/ or paraplegia/ or Pott's paraplegia/ 17724

63 parapleg*.mp. 25381

64 spastic quadriplegia/ or quadriplegia/ 8584

65 quadripleg*.mp. 11391

66 hearing impaired person/ 3598

67 hearing impairment/ 22617

68 hearing loss.mp. 89710

69 deaf*.mp. 56954

70 visually impaired person/ 3034

71 congenital blindness/ or blindness/ or partial blindness/ 22202

72 (blind* not (double blind or single blind or blind study)).mp. 187743

73 visual disorder/ 32214

74 developmental disorder/ 0

75 walking difficulty/ 0

76 mobility limitation/ 5692

77 wheelchair/ 5907

78 wheelchair.mp. 9032

79 self help device/ 6388

80 spinal cord injury/ 48300

81 spinal cord injur*.mp. 66266

82 cerebral palsy/ 25628

83 cerebral palsy.mp. 35919

84 spinal dysraphism/ 7024

85 spina bifida.mp. 10541

86 multiple sclerosis/ 68792

87 multiple sclerosis.mp. 111879

88 amyotrophic lateral sclerosis/ 26243

89 Amyotrophic Lateral Sclerosis.mp. 39448

90 als.mp. 82266

91 Brain injuries, traumatic/ 17220

92 traumatic brain injury.mp. 56848

93 Duchenne muscular dystrophy/ or muscular dystrophy/ 22453

94 muscular dystrophy.mp. 31515

95 Attention Deficit Disorder with Hyperactivity/ 38423

96 adhd.mp. 38345

97 attention deficit disorder.mp. 39416

98 Muscular Atrophy, Spinal/ 5591

99 spinal muscular atrophy.mp. 7892

100 muscle disease/ 0

101 myopathy.mp. 27792

102 autism/ 29083

103 ASD.mp. 43562

104 autism.mp. 77661

105 Down Syndrome/ 27696

106 down syndrome.mp. 33833

107 peripheral neuropathy/ 25904

108 peripheral neuropathy.mp. 26545

109 ((vision or visual* or hearing or mental or psychiatric or function* or activity or mobility or cognitive or intellectual) adj2 (limitation or limited or impair* or disab*)).mp. 423702

110 undocumented immigrant/ or immigrant/ or refugee camp/ or refugee/ or unaccompanied refugee minor/ or refugee crisis/ or migrant/ or long distance migrant/ or forced migrant/ or migrant worker/ or short distance migrant/ 45694

111 "Emigrants and Immigrants"/ or "Emigration and Immigration"/ or "Refugees"/ or "Transients and Migrants"/ or "Undocumented Immigrants"/ or "adopted country".mp. or alien.mp. or aliens.mp. or asylee.mp. or asylees.mp. or asylum.mp. or "au pairs".mp. or "au pair".mp. or "birth abroad".mp. or "birth countries".mp. or "birth country".mp. or "birth overseas".mp. or "born abroad".mp. or "born overseas".mp. or "border communities".mp. or "border community".mp. or "border health".mp. or "civil surgeon".mp. or "civil surgeons".mp. or citizenship.mp. or "countries of birth".mp. or "country of birth".mp. or "country of destination".mp. or "countries of origin".mp. or "country of origin".mp. or "customs and border protection".mp. or "deferred action for childhood arrivals".mp. or daca.mp. or "department of homeland security".mp. or deport.mp. or deportation.mp. or deportations.mp. or deported.mp. or "destination countries".mp. or "destination country".mp. or "diasporic communities".mp. or "diasporic community".mp. or "displaced person".mp. or "displaced persons".mp. or "displaced children".mp. or "displaced families".mp. or "displaced individuals".mp. or "displaced men".mp. or "displaced patients".mp. or "displaced people".mp. or "displaced peoples".mp. or "displaced women".mp. or "internationally displaced".mp. or "duration of residence".mp. or "ellis island".mp. or emigrate.mp. or emigrated.mp. or emigration.mp. or emigrant.mp. or emigrants.mp. or emigrating.mp. or "exchange student".mp. or "exchange students".mp. or "exchange visitor".mp. or "exchange visitors".mp. or exile.mp. or exiled.mp. or exiles.mp. or expatriation.mp. or expatriate.mp. or expatriates.mp. or expatriated.mp. or "family reunification".mp. or "family reunifications".mp. or "first generation".mp. or "first generations".mp. or "2.5 generation".mp. or "2.5 generations".mp. or "second generations".mp. or "third generation".mp. or "third generations".mp. or "mixed generation".mp. or "foreign birth".mp. or foreign-born.mp. or "foreign brides".mp. or "foreign citizen".mp. or "foreign citizens".mp. or "foreign citizenship".mp. or "foreign families".mp. or "foreign family".mp. or "foreign children".mp. or "foreign men".mp. or "foreign minor".mp. or "foreign minors".mp. or "foreign female".mp. or "foreign females".mp. or "foreign individuals".mp. or "foreign male".mp. or "foreign males".mp. or "foreign patient".mp. or "foreign patients".mp. or "foreign people".mp. or "foreign person".mp. or "foreign persons".mp. or "foreign resident".mp. or "foreign residents".mp. or "foreign national".mp. or "foreign nationals".mp. or "foreign nationalities".mp. or "foreign nationality".mp. or "foreign population".mp. or "foreign populations".mp. or "foreign woman".mp. or "foreign women".mp. or "foreigner".mp. or "foreigners".mp. or "green card".mp. or "green cards".mp. or "guest worker".mp. or "guest workers".mp. or "h 1b".mp. or "host countries".mp. or "host country".mp. or "host societies".mp. or "host society".mp. or immigrant.mp. or immigrants.mp. or immigrate.mp. or immigrated.mp. or immigrates.mp. or immigrating.mp. or immigration.mp. or "intercountry adoption".mp. or "intercountry adoptions".mp. or "international adoption".mp. or "international adoptions".mp. or "international student".mp. or "international students".mp. or "length of residence".mp. or "length of residency".mp. or "maternal nativity".mp. or migrant.mp. or migrants.mp. or migration.mp. or "migrated children".mp. or nativity.mp. or naturalization.mp. or naturalized.mp. or "new countries".mp. or "new country".mp. or "new entrant".mp. or "new entrants".mp. or "new settler".mp. or "new settlers".mp. or "non citizen".mp. or "non citizens".mp. or noncitizen.mp. or noncitizens.mp. or "non national".mp. or "non nationals".mp. or nonnational.mp. or nonnationals.mp. or nonnative.mp. or nonnatives.mp. or "non native".mp. or "non natives".mp. or "non refoulement".mp. or nonrefugee.mp. or nonrefugees.mp. or "overseas birth".mp. or "overseas born".mp. or "permanent residence".mp. or "permanent residency".mp. or "permanent resident".mp. or "permanent residents".mp. or "port health".mp. or postmigration.mp. or premigration.mp. or "receiving countries".mp. or "receiving country".mp. or "receiving societies".mp. or "receiving society".mp. or refugee.mp. or refugees.mp. or "region of origin".mp. or "regions of origin".mp. or "resettlement countries".mp. or "resettlement country".mp. or "seasonal farmworkers".mp. or "seasonal farm worker".mp. or "seasonal farm workers".mp. or "seasonal farmworker".mp. or "seasonal worker".mp. or "seasonal workers".mp. or "stateless people".mp. or "stateless person".mp. or "stateless persons".mp. or "stateless children".mp. or "stateless individuals".mp. or "stateless patients".mp. or "temporary protected status".mp. or "transnational community".mp. or "unaccompanied child".mp. or "unaccompanied children".mp. or "unaccompanied minor".mp. or "unaccompanied minors".mp. or undocumented.mp. or UNHCR.mp. or visa.mp. or visas.mp. or "voluntary return".mp. or xenophobia.mp. or xenophobic.mp. 582968

112 58 or 59 or 60 or 61 or 62 or 63 or 64 or 65 or 66 or 67 or 68 or 69 or 70 or 71 or 72 or 73 or 74 or 75 or 76 or 77 or 78 or 79 or 80 or 81 or 82 or 83 or 84 or 85 or 86 or 87 or 88 or 89 or 90 or 91 or 92 or 93 or 94 or 95 or 96 or 97 or 98 or 99 or 100 or 101 or 102 or 103 or 104 or 105 or 106 or 107 or 108 or 109 1648300

113 40 or 44 or 54 or 57 or 110 or 111 or 112 3242573

114 mental health/ 75947

115 mental health.mp. 342068

116 mental disease/ 0

117 Mental disorder*.mp. 243721

118 mental patient/ 6488

119 mental* illness*.mp. 47752

120 Anxiety/ 126022

121 anxiety.mp. 374430

122 "mixed anxiety and depression"/ or Generalized Anxiety Disorder-2/ or anxiety/ or anxiety neurosis/ or anxiety disorder/ or Generalized Anxiety Disorder-7/ or generalized anxiety disorder/ 159390

123 late life depression/ or major depression/ or chronic depression/ or atypical depression/ or long term depression/ or adolescent depression/ or depression/ or bipolar depression/ 220805

124 depression.mp. 572292

125 suicide attempt/ or suicide/ 63551

126 suicid*.mp. 131078

127 suicidal behavior/ 0

128 posttraumatic stress disorder/ 47001

129 PTSD.mp. 40703

130 post-traumatic stress.mp. 24192

131 cptsd.mp. 570

132 Alzheimer disease/ 137797

133 Alzheimers.mp. 204344

134 dementia/ 69537

135 Dementia.mp. 188191

136 bipolar I disorder/ or bipolar mania/ or bipolar disorder/ or bipolar II disorder/ 48152

137 Bipolar.mp. 100644

138 bpd.mp. 14781

139 borderline personality.mp. 11930

140 multiple personality/ or dissociative disorder/ 5091

141 dissociative identity.mp. 1208

142 simple schizophrenia/ or latent schizophrenia/ or catatonic schizophrenia/ or schizophrenia spectrum disorder/ or treatment-resistant schizophrenia/ or paranoid schizophrenia/ or schizophrenia/ 122588

143 Schizophrenia.mp. 174304

144 Altered Perception Syndrome.mp. 2

145 Psychosis Spectrum Syndrome.mp. 1

146 Neuro-Emotional Integration Disorder.mp. 3

147 drug dependence/ or addiction medicine/ or cannabis addiction/ or morphine addiction/ or opium addiction/ or addiction/ or behavioral addiction/ or opiate addiction/ or pathological gambling/ or gambling/ or alcohol abuse/ or alcohol rehabilitation/ 217487

148 addiction.mp. 70066

149 gambl*.mp. 14152

150 "substance use".mp. 64320

151 substance abuse.mp. 62212

152 alcoholism.mp. 93696

153 alcoholic.mp. 119660

154 alcohol dependence.mp. 11538

155 cocaine.mp. 49628

156 cigarette*.mp. 93964

157 heroin.mp. 22086

158 114 or 115 or 116 or 117 or 118 or 119 or 120 or 121 or 122 or 123 or 124 or 125 or 126 or 127 or 128 or 129 or 130 or 131 or 132 or 133 or 134 or 135 or 136 or 137 or 138 or 139 or 140 or 141 or 142 or 143 or 144 or 145 or 146 or 147 or 148 or 149 or 150 or 151 or 152 or 153 or 154 or 155 or 156 or 157 2248322

159 patient navigation.mp. 2317

160 navigation services.mp. 355

161 patient coordination.mp. 43

162 care coordination.mp. 7271

163 patient support services.mp. 50

164 159 or 160 or 161 or 162 or 163 9656

165 113 and 158 and 164 560

# EBSCOHost CINAHL

Top of Form

| **#** | **Query** |
| --- | --- |
| S105 | S76 AND S100 AND S104 |
| S104 | S101 OR S102 OR S103 |
| S103 | care coordination OR patient support services |
| S102 | navigation services |
| S101 | (MH "Patient Navigation") OR "patient navigation" |
| S100 | S77 OR S78 OR S79 OR S80 OR S81 OR S82 OR S83 OR S84 OR S85 OR S86 OR S87 OR S88 OR S89 OR S90 OR S91 OR S92 OR S93 OR S94 OR S95 OR S96 OR S97 OR S98 OR S99 |
| S99 | gambl* OR substance abuse OR "substance use" OR alcoholic |
| S98 | (MH "Behavior, Addictive") OR (MH "Substance Abuse") OR (MH "Heroin") OR "addiction" OR (MH "Alcoholism") |
| S97 | (MH "Behavior, Addictive") OR (MH "Substance Abuse") OR (MH "Heroin") OR "addiction" OR (MH "Alcoholism") |
| S96 | Neuro-Emotional Integration Disorder.mp |
| S95 | (MH "Schizophrenia") |
| S94 | schizophrenia |
| S93 | dissociative identity |
| S92 | (MH "Dissociative Identity Disorder") |
| S91 | bpd OR borderline personality |
| S90 | "bipolar" |
| S89 | (MH "Dementia") OR "dementia" OR (MH "Dementia Patients") |
| S88 | Alzheimers |
| S87 | (MH "Alzheimer's Disease") |
| S86 | post traumatic stress |
| S85 | cptsd |
| S84 | "ptsd" |
| S83 | (MH "Suicide") OR "suicid*" OR (MH "Suicidal Ideation") OR (MH "Suicide Prevention") |
| S82 | "depressive disorder" |
| S81 | (MH "Depression") OR "depression" |
| S80 | (MH "Anxiety") OR "anxiety" OR (MH "Generalized Anxiety Disorder") OR (MH "Social Anxiety Disorders") |
| S79 | mental illness* |
| S78 | mental health OR mental disorder* |
| S77 | (MH "Mental Health") OR (MH "Mental Disorders") |
| S76 | S16 OR S21 OR S31 OR S34 OR S71 OR S75 |
| S75 | S72 OR S73 OR S74 |
| S74 | "adopted country" OR alien OR aliens OR asylee OR asylees OR asylum OR "au pairs" OR "au pair" OR "birth abroad" OR "birth countries" OR "birth country" OR "birth overseas" OR "born abroad" OR "born overseas" OR "border communities" OR "border community" OR "border health" OR "civil surgeon" OR "civil surgeons" OR citizenship OR "countries of birth" OR "country of birth" OR "country of destination" OR "countries of origin" OR "country of origin" OR "customs and border protection" OR "deferred action for childhood arrivals" OR daca OR "department of homeland security" OR deport OR deportation OR deportations OR deported OR "destination countries" OR "destination country" OR "diasporic communities" OR "diasporic community" OR "displaced person" OR "displaced persons" OR "displaced children" OR "displaced families" OR "displaced individuals" OR "displaced men" OR "displaced patients" OR "displaced people" OR "displaced peoples" OR "displaced women" OR "internationally displaced" OR "duration of residence" OR "ellis island" OR emigrate OR emigrated OR emigration OR emigrant OR emigrants OR emigrating OR "exchange student" OR "exchange students" OR "exchange visitor" OR "exchange visitors" OR exile OR exiled OR exiles OR expatriation OR expatriate OR expatriates OR expatriated OR "family reunification" OR "family reunifications" OR "first generation" OR "first generations" OR "2.5 generation" OR "2.5 generations" OR "second generations" OR "third generation" OR "third generations" OR "mixed generation" OR "foreign birth" OR foreign-born OR "foreign brides" OR "foreign citizen" OR "foreign citizens" OR "foreign citizenship" OR "foreign families" OR "foreign family" OR "foreign children" OR "foreign men" OR "foreign minor" OR "foreign minors" OR "foreign female" OR "foreign females" OR "foreign individuals" OR "foreign male" OR "foreign males" OR "foreign patient" OR "foreign patients" OR "foreign people" OR "foreign person" OR "foreign persons" OR "foreign resident" OR "foreign residents" OR "foreign national" OR "foreign nationals" OR "foreign nationalities" OR "foreign nationality" OR "foreign population" OR "foreign populations" OR "foreign woman" OR "foreign women" OR foreigner OR foreigners OR "green card" OR "green cards" OR "guest worker" OR "guest workers" OR "h 1b" OR "host countries" OR "host country" OR "host societies" OR "host society" OR immigrant OR immigrants OR immigrate OR immigrated OR immigrates OR immigrating OR immigration OR "intercountry adoption" OR "intercountry adoptions" OR "international adoption" OR "international adoptions" OR "international student" OR "international students" OR "length of residence" OR "length of residency" OR "maternal nativity" OR migrant OR migrants OR migration OR "migrated children" OR nativity OR naturalization OR naturalized OR "new countries" OR "new country" OR "new entrant" OR "new entrants" OR "new settler" OR "new settlers" OR "non citizen" OR "non citizens" OR noncitizen OR noncitizens OR "non national" OR "non nationals" OR nonnational OR nonnationals OR nonnative OR nonnatives OR "non native" OR "non natives" OR "non refoulement" OR nonrefugee OR nonrefugees OR "overseas birth" OR "overseas born" OR "permanent residence" OR "permanent residency" OR "permanent resident" OR "permanent residents" OR "port health" OR postmigration OR premigration OR "receiving countries" OR "receiving country" OR "receiving societies" OR "receiving society" OR refugee OR refugees OR "region of origin" OR "regions of origin" OR "resettlement countries" OR "resettlement country" OR "seasonal farmworkers" OR "seasonal farm worker" OR "seasonal farm workers" OR "seasonal farmworker" OR "seasonal worker" OR "seasonal workers" OR "stateless people" OR "stateless person" OR "stateless persons" OR "stateless children" OR "stateless individuals" OR "stateless patients" OR "temporary protected status" OR "transnational community" OR "unaccompanied child" OR "unaccompanied children" OR "unaccompanied minor" OR "unaccompanied minors" OR undocumented OR UNHCR OR visa OR visas OR "voluntary return" OR xenophobia OR xenophobic |
| S73 | (MH "Refugees") |
| S72 | (MH "Emigration and Immigration") OR (MH "Immigrants") OR "emigrants" |
| S71 | S35 OR S36 OR S37 OR S38 OR S39 OR S40 OR S41 OR S42 OR S43 OR S44 OR S45 OR S46 OR S47 OR S48 OR S49 OR S50 OR S51 OR S52 OR S53 OR S54 OR S55 OR S56 OR S57 OR S58 OR S59 OR S60 OR S61 OR S62 OR S63 OR S64 OR S65 OR S66 OR S67 OR S68 OR S69 OR S70 |
| S70 | ((vision OR visual* OR hearing OR mental OR psychiatric OR function* OR activity OR mobility OR cognitive OR intellectual ) N2 (limitation OR limited OR impair* OR disab* )) |
| S69 | peripheral neuropathy |
| S68 | (MH "Peripheral Nervous System Diseases") OR "peripheral nervous system disease" |
| S67 | (MH "Down Syndrome") OR "down syndrome" |
| S66 | (MH "Autism Spectrum Disorder") OR (MH "Asperger Syndrome") OR "autism" |
| S65 | "myopathy" |
| S64 | adhd OR attention deficit disorder |
| S63 | (MH "Attention Deficit Hyperactivity Disorder") OR (MH "Neurodiversity") |
| S62 | (MH "Muscular Dystrophy+") |
| S61 | (MH "Brain Injuries") OR "traumatic brain injury" |
| S60 | als |
| S59 | (MH "Amyotrophic Lateral Sclerosis") OR "amyotrophic lateral sclerosis" |
| S58 | (MH "Multiple Sclerosis") OR "multiple sclerosis" |
| S57 | (MH "Spina Bifida") OR "spina bifida" |
| S56 | cerebral palsy |
| S55 | (MH "Cerebral Palsy") |
| S54 | (MH "Spinal Cord Injuries") OR (MH "Spinal Cord Diseases") |
| S53 | "self help devices" |
| S52 | "self help devices" |
| S51 | wheelchair |
| S50 | (MH "Wheelchairs") |
| S49 | "mobility limitation" |
| S48 | (MH "Ambulation: Wheelchair (Iowa NOC)") |
| S47 | (blind* NOT ("double blind" OR "single blind" OR "blind study" )) |
| S46 | (MH "Developmental Disabilities") OR (MH "Intellectual Disability") |
| S45 | (MH "Vision Disorders") |
| S44 | (MH "Deaf-Blind Disorders") OR (MH "Blindness") OR (MH "Vision, Subnormal") |
| S43 | (MH "Persons with Visual Disabilities") OR "visually impaired" |
| S42 | (MH "Hearing Disorders") OR (MH "Hearing Aids") OR (MH "Persons with Hearing Disabilities") OR (MH "Hearing Loss, Hidden") OR "hearing impaired" |
| S41 | (MH "Deafness") OR (MH "Deaf-Blind Disorders") OR "deaf" |
| S40 | quadripleg* |
| S39 | "paraplegic" |
| S38 | amputee* |
| S37 | (MH "Amputees") |
| S36 | disability |
| S35 | (MH "Persons with Disabilities") |
| S34 | S32 OR S33 |
| S33 | (aborig* OR indigen* OR "first nation*" OR first-nation* OR "first peoples" OR "native people*" OR (alaska* N3 native* ) OR inuit* OR inu#k OR Eskimo* OR metis OR (native* N3 america* ) OR (hawai* N2 native* ) OR "pacific islander*" OR ("pacific island*" N3 native* ) OR "america* indian*" OR amerindian* OR "Torres Strait Island*" OR Maori* ) |
| S32 | (MH "Indigenous Women") OR (MH "Indigenous Health") OR (MH "Indigenous Peoples") OR (MH "Health Services, Indigenous") OR (MH "Native Hawaiians") OR (MH "Alaska Natives") OR (MH "Aboriginal Canadians") OR (MH "Native Americans") OR (MH "Aboriginal Australians") OR (MH "Apache (Native American People)") |
| S31 | S22 OR S23 OR S24 OR S25 OR S26 OR S27 OR S28 OR S29 OR S30 |
| S30 | latino* OR latina* OR latine* OR latinx* |
| S29 | (MH "Hispanic Americans") OR (MH "Hispanic Caribbean Persons") |
| S28 | ((African N1 ("American* Canadian*" OR ancestry )) OR (Black N1 (m?n OR people OR person* OR student* OR wom?n )) OR Blacks ) |
| S27 | racialized OR racism |
| S26 | (((children OR communit* OR faculty OR men OR patien* OR people OR person OR persons OR women ) N2 colo#r ) OR ethnic OR ethnical OR ethnically OR ethnicized OR ethnicities OR ethnicism OR ethnicities OR ethnicity OR ethnics OR ethnic OR minorities OR minority OR race OR races OR racial OR racially ) |
| S25 | (MM "Black Canadians") OR (MH "Black Persons+") OR (MM "Black British Persons") OR (MM "Anti-Black Racism") OR (MH "People of Color+") OR (MM "African Americans") |
| S24 | (MH "Ethnic Groups") OR (MH "People of Color") OR (MH "Racialization") |
| S23 | (MH "Racism") OR (MH "Systemic Racism") OR (MH "Anti-Black Racism") OR (MH "Anti-Muslim Racism") |
| S22 | (MH "Race Relations") OR (MH "Anti-Asian Racism") |
| S21 | S17 OR S18 OR S19 OR S20 |
| S20 | (((economic OR economical OR economically ) N2 (distress OR distressed OR factors )) OR ((economically OR socially OR socioeconomically OR stability OR stable ) N1 disadvantaged ) OR income OR poverty OR ((social OR socially OR socioeconomic ) N2 (class OR determinants OR disparities OR disparity OR inequities OR inequity OR status ))) |
| S19 | poverty* OR impover* |
| S18 | (MH "Social Class") |
| S17 | (MH "Poverty") OR (MH "Homelessness") |
| S16 | S15 OR S14 OR S13 OR S12 OR S11 OR S10 OR S9 OR S8 OR S7 OR S6 OR S5 OR S4 OR S3 OR S2 OR S1 |
| S15 | two spirit |
| S14 | LGB* OR GLB* OR 2SLGB* |
| S13 | intersex |
| S12 | (MH "Intersex Persons") OR (MH "Intersexuality") |
| S11 | asexual* OR ace-spectrum |
| S10 | (MH "Asexual Persons") OR (MH "Asexuality") |
| S9 | transgender* OR transmasc* OR transfem* OR transm?n OR trans m?n OR transwom?n OR trans wom?n OR ( ((gender OR sex) N2 (change* OR confirmation OR disorder OR divers* OR dysphoria OR identity OR minorit* OR nonconforming OR non-conforming OR reassignment OR surgeries OR surgery)) ) OR genderqueer OR non-binary OR nonbinary OR genderfluid |
| S8 | (MH "Transsexual Persons") |
| S7 | (MM "Transgender Persons") OR (MM "Trans Women") OR (MM "Trans Men") |
| S6 | (MM "Non-Monogamous Relationship") |
| S5 | bisexual* |
| S4 | "pansexual" |
| S3 | (MH "Bisexual Persons") OR (MH "Bisexuality") |
| S2 | XB lesbian* OR XB gay* OR XB queer OR XB homosexual* OR XB MSM OR XB males who have sex with males OR XB men who have sex with men OR XB women who have sex with women OR XB nonheterosex* OR XB non-heterosex* OR XB polysexual* OR XB polyamor* |
| S1 | (MM "Queer Persons") OR (MM "Black LGBTQ+ Persons") OR (MH "LGBTQ+ Persons+") |

Bottom of Form
