# Supplementary material for: Pathways to inclusion: A scoping review exploring equity, diversity, and inclusion in mental health and addictions patient navigation programs
Source: PLOS Ment Health. 2026 Jun 25;3(6):e0000557. doi: 10.1371/journal.pmen.0000557 (PMC13298921; doi:10.1371/journal.pmen.0000557)
Supplement: Attachment — Submitted filename: 20260508 PLOS Mental Health_Response.docx [file pmen.0000557.s004.docx]

Dear Editors and Reviewers,

We deeply appreciate the feedback provided on our manuscript, “Pathways to inclusion: A scoping review exploring equity, diversity, and inclusion in mental health and addictions patient navigation programs.” We extend our sincerest gratitude for the time and consideration given to this manuscript, and for the reviewer’s thoughtful suggestions.

Please find our responses to the reviewers’ comments below, including how and where the text was modified. Responses are organized sequentially and by reviewer. An updated manuscript with tracked changes edits has also been attached.

Thank you,

The authors

**REVIEWER 1 COMMENTS:**

1. **The introduction focuses primarily on individual level barriers to care. Structural and systemic barriers are only briefly mentioned at the end of 1.2. It would be helpful to further explicate the nature of structural barriers and their disparate impacts on EDCs in the introduction, to better set up the rationale for the scoping review.**

RESPONSE: Thank you for highlighting the importance of expanding on structural and systemic barriers to rationalize this scoping review. We have included additional examples to capture this on pages 4-5:

“Sundareswaran et al. [21] identified language discordance as a hurdle to care for newcomers, including when booking appointments over the phone, understanding medical vocabulary, and accessing translated written documents. Newcomers’ mental healthcare experiences are directly impacted by the lack of funding for and absence of adequate professional interpretation services [22]. Healthcare funding schemes further disadvantage low-income and uninsured individuals, for whom high out-of-pocket costs for certain mental health services or prescription medications create additional stress and uncertainty [21,23,24]. Competing priorities, such as employment responsibilities, also often take precedence over seeking care [21,22,25]. Colonial policies continue to diminish Indigenous health needs through the underdevelopment and underfunding of health centers in remote communities and on reserves. As a result, the far distances to medical facilities and limited infrastructure make it challenging to receive timely care [25].”

2. **In section 1.3. there is a brief description of navigator programs. It would be helpful to explain this description to describe their core components and research base, before introduction the special focus on navigation with EDCs. This would distinguish what is know about navigation to MHA treatments generally and the unique contribution of the current study to the population of interest.**

RESPONSE: Thank you for directing our attention to the need to further explain the core components of navigator programs and the existing research base. We have addressed this through the addition of the following text (pages 5-6):

Core components of navigator programs:

- “Navigation services have expanded widely since to support patients with identifying appropriate supports in complex care systems for a variety of conditions (e.g., dementia [28], diabetes [29], HIV [30]).”
- “Barrier reduction, client-centered support, and integration of care are core themes seen across MHA navigation programs [31]. There are various models of care used, including professional navigation by clinicians or trained staff and lay navigation by peers. Delivery methods offered may include virtual (i.e., email, text message, phone), in-person, or a combination of both [32, 33].”

Research base:

- “The evolution of navigation services has brought with it a growing base of scientific literature [35]. Studies assessing MHA navigation have demonstrated the substantial promise of these programs in improving care experiences, participation, and outcomes (e.g., reduced number of mental and physical health problems, improved quality of life) [33]. However, recent reviews have been largely focused on cancer care [36,37]. For EDCs who face unique barriers in the healthcare system, the approaches used in MHA navigation may require particular consideration. There has been no scoping review conducted to date synthesizing the application of MHA navigation services to best support individuals belonging to EDCs. This is needed to determine how such programs can be developed and improved to alleviate barriers to appropriate care and better serve the needs of these communities.”

3. **In section 2.2. Could an article be included if it did not focus on an EDC? How did the search and review process disentangle programs for EDC populations from the general population or another non-EDC special population? The search and inclusion / exclusion process seems unclear.**

RESPONSE: We have added text to clarify the inclusion/exclusion process on page 7: “Articles that were not fully focused on an EDC defined in this study (e.g., findings pertaining to EDCs reported as a sub-group analysis rather than being the focus of the study) were also excluded.”

Papers that did not specifically focus on low-income individuals, racial and ethnic minorities, members of 2SLGBTQI+ communities, people with disabilities, Indigenous Peoples, and/or newcomers were excluded during the screening process.

As shown in the S2 supplementary file, the search strategy included terms to identify these EDCs (search terms 1-127). We have added examples of the key search terms used in section 2.3 (page 8) to clarify the search process: “Key terms used to develop the search include: 1) equity-deserving communities (e.g., “sexual and gender minorities”, “racial groups”, “Indigenous Peoples”) AND 2) mental health (e.g., “mental disorders”, “substance use”, “addiction”) AND 3) navigation programs (e.g., “patient navigation”, “navigation services”).”

4. **Section 2.3. The search methodology seems problematic, this reviewer is uncertain if the selected terms are sufficient to identify relevant studies. Note: The reviewer did examine the search terms in the supplemental material. Barriers, facilitators, kinship navigation and family navigation were not included which seems to have resulted in some missed studies. The terms also seem to focus on disease and disability. It could be helpful to include terms like health promotion, equity, etc to find studies that describe navigation outcomes.**

RESPONSE: We appreciate the reviewer’s concern regarding the search methodology. The search strategy was carefully developed by the research team and an expert academic librarian, with terms selected to specifically capture literature related to the objective of this review. Barriers, facilitators, health promotion, and equity terms were not included in our search as we were not looking for outcome-related studies. Instead, we wanted to identify navigation approaches/practices for supporting EDCs. Therefore, including these additional terms would create noise in the search results and broaden the search beyond the scope of this paper.

The terms kinship navigation and family navigation were tested during the development of the search strategy and did not yield any relevant results. Additionally, these navigation types were captured by the general “navigation services” search term. Examination of the studies identified by our search revealed results related to kinship navigation and family navigation. In preparing our response to this comment, we again tested the search with the academic librarian to see if any new studies come up when these two terms are added. This search yielded 6 new articles that were not identified by our original search; however, none of these articles met our criteria for inclusion.

The search terms focused on disease or disability that are included were meant to comprehensively capture studies on people with disabilities, as this is one of the EDCs of focus in this paper.

5. **Section 2.4. The reporting of study selection is not replicable. Were structured decision making tools created for this process? Its not clear how systematic the selection of studies were and what decision rules were when a paper included some EDC but focused fully on them.**

RESPONSE: Thank you for bringing up the need for clarification of the study selection process. We apologize for missing these details. We have added the following text to pages 8-9 to address this concern (additions in red):

“All identified articles were imported into Covidence for de-duplication and screening. The inclusion and exclusion criteria were carefully applied to decide on whether a study should be included. Only studies meeting all inclusion criteria were included.

Two independent reviewers (AC, RS) first conducted a pilot title and abstract screening with 20 articles, with each reviewer using a structured Excel spreadsheet to indicate each study’s citation details and the reason for inclusion or exclusion. A discussion was then held to reach consensus on conflicts based on comparison of Excel spreadsheets and ensure reviewers had an aligned understanding of the eligibility criteria before continuation of the screening process. Following this, the remaining titles and abstracts were screened by two reviewers (AC, RS), with a third reviewer (RM) available to resolve conflicts throughout. After completion of title and abstract screening, two team members (AC, RS) conducted a pilot full-text review with five articles. Each reviewer again used a structured Excel spreadsheet to indicate each study’s citation details and the reason for inclusion or exclusion. A discussion was held to reach consensus before the remaining full-texts were reviewed by the two team members (AC, RS). A third reviewer (RM) resolved any conflicts that arose.”

In response to another comment above, the eligibility criteria was also clarified in section 2.2 (page 7) to confirm that articles had to be focused fully on an EDC to be included.

6. **Section 2.6. Describe what MAXQDA is for the reader and how it helped supported study implementation.**

RESPONSE: We appreciate the suggestion to add an explanation of MAXQDA for readers who may not be familiar with this software. The following text has been added on pages 9-10 to briefly describe how MAXQDA supported this study:

“MAXQDA is a software program used for coding in qualitative data analysis. This software supported the visualization and identification of key data in the included studies, which allowed for synthesis into the findings of this review [46].”

7. **Results Table 1 doesn't clearly identify the MHA need or population of interest for all studies. There are a few key studies that are important to the research question that are missing, raising some critical questions about the thoroughness of the search and inclusion process.**

RESPONSE: Thank you for your feedback on how we can improve Table 1. We have added a column for the MHA concern of focus in each study to this Results table. The population of interest for each study is shown through the “EDCs Included” column. The heading for this column has been changed to “EDCs of Focus” to make this clearer.

The study search and inclusion processes were conducted carefully and thoroughly; the authors welcome examples of key studies mentioned by the reviewer to investigate why they may have been missed.

8. **Table 2 provides some details about the nature of the findings, but it is difficult to extrapolate some of them into specific practice behaviors. A more detailed reporting of the implications for practice, as it was reported by the study authors, would be helpful.**

RESPONSE: Thank you for highlighting the need for a more detailed reporting of practice implications in Table 2. We have edited the text throughout this table accordingly to ensure there are actionable steps described as reported by the study authors. In addition, the subtitle for column 2 of Table 2 was edited from “Strategies to Improve MHA Navigation for EDCs” to “Key Findings and Strategies to Improve MHA Navigation for EDCs”. We hope this will more clearly indicate that main findings from each study are meant to be shown through this table (e.g., barriers to care) in addition to the specific approaches of navigation.

9. **The findings from section 3 largely mirror what is known from navigation research with populations other than EDCs. It would be helpful to distinguish the unique barriers, facilitators, and interventions for EDC's and describe how they are similar or different to non EDC's. As it reads currently the strategies for working with EDC's appear to be universal. The section might also be better organized into sub themes within each of the 3 categories of findings to make the presentation of information more interpretable. For example, what are some indigenous, veteran, or LGBTQ specific methods to promote service access? What unique barriers to they experience that differ from non EDC populations? works within or across populations? What are the strengths and haps gaps within the research for each EDC that was studies?**

RESPONSE: Thank you for your suggestions on how we can better present the findings from section 3. As per your feedback, we have added subtheme headings under each theme to improve the organization of findings:

- The subtheme headings “Practical barriers”, “Scheduling conflicts”, “Systemic barriers”, and “Barriers to accessing navigation services” were added to 3.2.1.
- The subtheme headings “Barriers and facilitators to MHA service utilization” and “Barriers and facilitators to navigation program utilization” were added to 3.2.2.
- The subtheme headings “Navigation support for access to social programming”, “Instrumental supports provided by navigators”, “Engaging circles of support to enable equitable care”, “Psychological supports provided by navigators”, and “Optimizing the patient experience” were added to 3.2.3.
- The subtheme headings “Operational practices in navigation programs”, “Equity-driven improvement models”, “Accessible navigation schedules”, “Accessible navigation delivery modes”, and “Socially-inclusive navigation practices” were added to 3.2.4.

We recognize that the findings presented are not segregated by each equity-deserving group. This was intentional based on the extracted data as well as the aim of this study, which was to more broadly describe approaches in MHA navigation among EDCs. Majority of the discussed approaches were agreed upon across multiple included articles; therefore, segregating the results by EDC may risk inaccuracy and exclusion of certain groups. Moreover, drawing inferences about specific groups may be premature based on the nature and limited volume of literature to date. Given that social identities often overlap, individuals may also belong to more than one EDC.

To more clearly describe the unique barriers experienced by the EDCs of focus in this paper compared to non-EDC populations, we have also expanded section 1.2 per our response to comment #1. As this review describes navigation practices for EDCs specifically and the included studies did not offer comparisons to non-EDCs, we chose not to discuss navigation work in dominant populations; this may extend beyond the scope of this review. Nonetheless, we recognize that this is an important avenue of research. We have added the lack of comparison to non EDCs and segregation by equity-deserving group as limitations on pages 38-39 to address this concern:

“This review also did not segregate results by each EDC given that approaches largely overlapped across all included studies and individuals often identify with more than one social group. Assessing differences between specific equity-deserving groups, as well as in comparison with other dominant groups, may be a focus of future research.”

In addition, we understand that the strategies presented may be universally beneficial. However, these approaches/features are not always consistently present all together within individual programs. When working with EDCs, it is important that navigation programs strive to intentionally incorporate as many of these approaches/features as possible to ensure these groups are not overlooked. We have added the following text to the discussion section (page 38) to clarify this:

“This scoping review synthesized promising navigation practices for supporting EDCs. While the discussed practices can be universally beneficial based on other literature on MHA navigation, they are not always consistently implemented together within individual programs [33]. This review points to the importance of recognizing the challenges uniquely faced by EDCs and intentionally incorporating as many of the identified practices/features as possible to make programs maximally accessible, responsive, and helpful for these groups. Overall, the findings underscore the importance of a deeper focus on equity and holistic support for marginalized populations in MHA navigation.”

**REVIEWER 2 COMMENTS:**

1. **The manuscript references Freeman’s conceptualization of navigation early in the introduction (lines 86–88). Including an explicit citation at this point would strengthen the conceptual grounding of the review and situate it more firmly within the foundational navigation literature.**

RESPONSE: Thank you for directing our attention to the missing citation for Freeman’s conceptualization of navigation. We have added an explicit citation to page 5 (line 99) to capture this.

2. **A brief rationale for the decision to include studies published from 2014 onward would improve transparency. Clarifying whether this reflects shifts in MHA navigation practice, policy developments, or increased attention to equity‑oriented navigation models would be helpful.**

RESPONSE: There was no publication year limit added to our search or used in our eligibility criteria for screening. Based on the other eligibility criteria listed in section 2.2, the studies that ended up being included in this scoping review happen to be from 2014 onward. This may reflect the evolution of navigation over the last decade, which was mentioned in section 1.3 (page 5). We have also added the following text to section 3.1.1 (page 11) to clarify: “The lack of studies from before 2014 reflect the expansion of patient navigation over recent years.”

3. **The ordering of studies in Table 1 is not immediately clear. A short explanation of the organizing logic (e.g., chronological, geographic, or thematic) would improve clarity.**

RESPONSE: Thank you for pointing out this missed detail in Table 1. The following text was added to the figure captions of Tables 1 and 2 to improve clarity: “(ordered alphabetically by author)”.

4. **One included source is described as a narrative review (p. 9). As narrative reviews are not typically considered primary sources within Arksey and O’Malley or JBI scoping review methodologies, the authors may wish to reconsider its inclusion or briefly justify its methodological fit within the data corpus.**

RESPONSE: We appreciate the reviewer's concern surrounding the inclusion of a narrative review in this paper. Upon reviewing the most updated JBI guidelines, we found confirmation in Chapter 10.2.4 of the JBI Manual for Evidence Synthesis that *any* existing literature can be included for the purposes of a scoping review. As secondary data sources are listed as examples of types of evidence sources that can be included in a scoping review, we have decided to keep the narrative review to maintain the greatest breadth of data possible.

5. **Several sections of the findings include interpretive or prescriptive language that appears more appropriate for the Discussion or Implications sections. For example, section 3.2.2 (line 263) begins with “Therefore, programs should…,” which reads as a recommendation rather than a descriptive synthesis. Reframing results sections to remain primarily descriptive, while reserving recommendations for later sections, would strengthen alignment with scoping review conventions.**

RESPONSE: Thank you for directing our attention to the language used in the findings that appears interpretive or prescriptive. We have rephrased the language to better reflect a descriptive synthesis and to ensure recommendations are reserved for the discussion section. We aimed to make clearer that the information is a direct reporting of findings from the included studies. Below is a list of changes made in red:

- ~~Therefore, programs should ensure that~~ Studies stress sources of support~~s~~ (e.g., supervisors, debrief sessions) and emotional regulation training as critical resources ~~available to~~ for navigators working with EDCs. (page 29)
- ~~For example, navigators should be~~ This includes ensuring navigators are made aware of important basic values when supporting EDCs, such as being recovery-focused, empowering, and accepting. (page 31)
- In addition, comprehensive training on skills to effectively work with EDCs ~~are~~ was noted as a critical component of program delivery, including reflective listening, motivational interviewing, using a strengths-based and harm reduction approach, facilitating interpersonal problem solving, and providing trauma-informed care. (page 31)
- Beyond direct patient interaction skills, EDC clients felt that navigators ~~should~~ must be primed to manage their own roles (i.e., setting boundaries and managing burnout) and have a strong understanding of the healthcare system and suitable resources ~~for EDCs~~. (pages 31-32)
- Moreover, Koob et al. [48] identified the importance of standardizing protocols based on existing evidence to better address EDCs’ specific needs, ~~could enhance~~ improve ~~both~~ individual outcomes, and allow for clearer, generalizable ~~standardized~~ evaluations across navigation programs ~~to~~ that can inform practice. Furthermore, studies note the importance of establishing the location in which navigation ~~can be~~is commenced ~~should be established~~ (e.g., during inpatient care, prior to discharge), taking into consideration the target population’s needs and where groups may be best engaged. (page 32)
- Studies also highlighted outreach to areas that do not offer navigation services ~~is also~~as a crucial strategy ~~to~~ for engaging EDCs. (page 32)
- The authors suggest that offering navigation during patients’ stays in these hospitals may improve reach and efficiency. (page 32)
- Koob et al. [48] proposed that efficiency can be further built into navigation programs by embracing emerging technologies to manage cases (e.g., using an integrated database to streamline data collection, merge personal health and referral data, and reduce errors from manual data entry), thereby enhancing navigators’ capacity to support vulnerable groups. (page 32)
- In addition to integrating new technologies, studies report the need for equity-driven improvements to existing referral systems and processes ~~are needed~~ to enhance support for EDCs. (page 32)
- Most importantly, van der Star et al. [59] stressed that it is crucial ~~that~~ for programs to continuously seek and implement patient feedback to ensure EDCs’ needs are comprehensively addressed. (page 33)
- Furthermore, ensuring navigation is linguistically accessible and tailored to individual circumstances ~~are~~ were also important considerations in the included studies, in recognition of the added social barriers EDCs face. (page 34)
- The sentence, “To offer culturally safe support, navigators should recognize that EDCs may have misconceptions about treatments or limited trust in service providers stemming from prior negative experiences in the healthcare system”, was moved from section 3.2.4 of the Results to the Discussion section (page 36).

6. **Given the strong equity focus of the review, a brief theoretical framing and/or positionality statement may help orient readers to the analytic lens informing the interpretation of the literature. This could further enhance transparency and contextual understanding.**

RESPONSE: Thank you for this thoughtful suggestion. We agree that a theoretical framing/positionality statement is important for enhancing transparency and contextual understanding. No specific theoretical framework guided this review. The authors are not comfortable individually disclosing their personal identities; however, recognizing the importance of reflexivity in qualitative work, a broader team positionality statement has been added on page 10:

“The authors recognize that positionality shapes the research process. The research team comprised members with different social identities, lived experiences, and professional backgrounds relevant to the populations of focus in this review. Representation of diverse genders, racial and ethnic backgrounds, ability statuses, and ages ensured that the search, study screening, and data extraction steps were informed by varying perspectives.”

7. **The discussion of limited generalizability to global populations (p. 31, lines 456–457) may not be necessary. The review is primarily descriptive in nature and does not appear to adopt a positivist orientation or make population‑level causal claims. Emphasizing transferability—rather than generalizability—may be more appropriate for a scoping review of this kind and more consistent with qualitative and equity‑oriented synthesis standards.**

RESPONSE: Thank you for pointing out the appropriateness of using transferability versus generalizability for this primarily descriptive scoping review. We have edited this limitation to emphasize transferability instead of generalizability on page 38 (red indicates the newly changed text):

“While this may limit the transferability of findings to other settings, the identified themes may remain useful in supporting MHA navigation programs identify the most suitable strategies to adopt to better support EDCs.”

8. **The review does not appear to attend to quantitative findings or their integration into the analysis. Clarifying whether quantitative evidence was encountered but not synthesized, or explicitly noting that the review prioritized qualitative and descriptive data, would improve methodological transparency.**

RESPONSE: This review prioritized descriptive data on approaches used in MHA navigation for EDCs. As suggested, we have explicitly noted this through the addition of the below text on page 39 to improve methodological transparency.

“As this review prioritized qualitative data to comprehensively describe approaches used in MHA navigation, further research assessing quantitative evidence is warranted to offer additional insight into outcomes.”
