# Supplementary material for: Pathways to inclusion: A scoping review exploring equity, diversity, and inclusion in mental health and addictions patient navigation programs
Source: PLOS Ment Health. 2026 Jun 25;3(6):e0000557. doi: 10.1371/journal.pmen.0000557 (PMC13298921; doi:10.1371/journal.pmen.0000557)
Supplement: Peer review history

# Peer review: Decision Letter 0

**DOI:** 10.1371/journal.pmen.0000557.r001

**Authors:** Karli Montague-Cardoso

30 Mar 2026

PMEN-D-26-00024

Pathways to inclusion: A scoping review exploring equity, diversity, and inclusion in mental health and addictions patient navigation programs

PLOS Mental Health

Dear Dr. Markoulakis,

Thank you for submitting your manuscript to PLOS Mental Health. I am very sorry for the delay.

After careful consideration of the reviewer reports, which we have now received, we feel that your paper has merit but does not fully meet PLOS Mental Health’s publication criteria as it currently stands. Therefore, we invite you to submit a revised version of the manuscript that addresses the points raised during the review process.

Please ensure that you address all of the comments, which you can find at the end of this email.

Please submit your revised manuscript by May 10 2026 11:59PM. If you will need more time than this to complete your revisions, please reply to this message or contact the journal office at mentalhealth@plos.org. When you're ready to submit your revision, log on to https://www.editorialmanager.com/pmen/ and select the 'Submissions Needing Revision' folder to locate your manuscript file.

Please include the following items when submitting your revised manuscript:

- A letter that responds to each point raised by the editor and reviewer(s). You should upload this letter as a separate file labeled 'Response to Reviewers'.
- A marked-up copy of your manuscript that highlights changes made to the original version. You should upload this as a separate file labeled 'Revised Manuscript with Track Changes'.
- An unmarked version of your revised paper without tracked changes. You should upload this as a separate file labeled 'Manuscript'.

Guidelines for resubmitting your figure files are available below the reviewer comments at the end of this letter.

As the corresponding author, your ORCID iD is verified in the submission system and will appear in the published article. PLOS supports the use of ORCID, and we encourage all coauthors to register for an ORCID iD and use it as well. Please encourage your coauthors to verify their ORCID iD within the submission system before final acceptance, as unverified ORCID iDs will not appear in the published article. *Only*  the individual author can complete the verification step; PLOS staff *cannot*  verify ORCID iDs on behalf of authors.

We look forward to receiving your revised manuscript.

Kind regards,

Karli Montague-Cardoso

Staff Editor

PLOS Mental Health

Journal Requirements:

1. Please provide a detailed online Financial Disclosure statement. This is published with the article. It must therefore be completed in full sentences and contain the exact wording you wish to be published.

a) State the initials, alongside each funding source, of each author to receive each grant. For example: “This work was supported by the National Institutes of Health (####### to AM; ###### to CJ) and the National Science Foundation (###### to AM).”

b) State what role the funders took in the study. If the funders had no role in your study, please state: “The funders had no role in study design, data collection and analysis, decision to publish, or preparation of the manuscript.”

For more information, please go to our submission guidelines:

https://journals.plos.org/mentalhealth/s/submission-guidelines#loc-financial-disclosure-statement

2. Please ensure that the funders and grant numbers match between the Financial Disclosure field and the Funding Information tab in your submission form. Note that the funders must be provided in the same order in both places as well.

3. Please update your online Competing Interests statement. If you have no competing interests to declare, please state: "The authors have declared that no competing interests exist."

4. Please provide separate main figure files in .tif or .eps format only and ensure that all files are under our size limit of 10MB.

For more information about how to convert your figure files please see our guidelines: https://journals.plos.org/mentalhealth/s/figures

5. If the reviewer comments include a recommendation to cite specific previously published works, please review and evaluate these publications to determine whether they are relevant and should be cited. There is no requirement to cite these works unless the editor has indicated otherwise.

Additional Editor Comments (if provided):

[Note: HTML markup is below. Please do not edit.]

Reviewers' comments:

Reviewer's Responses to Questions

**Comments to the Author**

1. Does this manuscript meet PLOS Mental Health’s publication criteria? Is the manuscript technically sound, and do the data support the conclusions? The manuscript must describe methodologically and ethically rigorous research with conclusions that are appropriately drawn based on the data presented.

Reviewer #1: Partly

Reviewer #2: Partly

**********

2. Has the statistical analysis been performed appropriately and rigorously?

Reviewer #1: N/A

Reviewer #2: N/A

**********

3. Have the authors made all data underlying the findings in their manuscript fully available (please refer to the Data Availability Statement at the start of the manuscript PDF file)?

The PLOS Data policy requires authors to make all data underlying the findings described in their manuscript fully available without restriction, with rare exception. The data should be provided as part of the manuscript or its supporting information, or deposited to a public repository. For example, in addition to summary statistics, the data points behind means, medians and variance measures should be available. If there are restrictions on publicly sharing data—e.g. participant privacy or use of data from a third party—those must be specified.

Reviewer #1: Yes

Reviewer #2: Yes

**********

4. Is the manuscript presented in an intelligible fashion and written in standard English?

PLOS Mental Health does not copyedit accepted manuscripts, so the language in submitted articles must be clear, correct, and unambiguous. Any typographical or grammatical errors should be corrected at revision, so please note any specific errors here.

Reviewer #1: Yes

Reviewer #2: Yes

**********

5. Review Comments to the Author

Please use the space provided to explain your answers to the questions above. You may also include additional comments for the author, including concerns about dual publication, research ethics, or publication ethics. (Please upload your review as an attachment if it exceeds 20,000 characters)

Reviewer #1: Thank you for the opportunity to review this manuscript, which addresses an important and understudied dimension of navigation research. The study is promising and, subject to significant revisions and extension of the literature search, may be suitable for publication. My recommendations to the authors are provided below:

The introduction focuses primarily on individual level barriers to care. Structural and systemic barriers are only briefly mentioned at the end of 1.2. It would be helpful to further explicate the nature of structural barriers and their disparate impacts on EDCs in the introduction, to better set up the rationale for the scoping review.

In section 1.3. there is a brief description of navigator programs. It would be helpful to explain this description to describe their core components and research base, before introduction the special focus on navigation with EDCs. This would distinguish what is know about navigation to MHA treatments generally and the unique contribution of the current study to the population of interest.

In section 2.2. Could an article be included if it did not focus on an EDC? How did the search and review process disentangle programs for EDC populations from the general population or another non-EDC special population? The search and inclusion / exclusion process seems unclear.

Section 2.3. The search methodology seems problematic, this reviewer is uncertain if the selected terms are sufficient to identify relevant studies. Note: The reviewer did examine the search terms in the supplemental material. Barriers, facilitators, kinship navigation and family navigation were not included which seems to have resulted in some missed studies. The terms also seem to focus on disease and disability. It could be helpful to include terms like health promotion, equity, etc to find studies that describe navigation outcomes.

Section 2.4. The reporting of study selection is not replicable. Were structured decision making tools created for this process? Its not clear how systematic the selection of studies were and what decision rules were when a paper included some EDC but focused fully on them.

Section 2.6. Describe what MAXQDA is for the reader and how it helped supported study implementation.

Results Table 1 doesn't clearly identify the MHA need or population of interest for all studies. There are a few key studies that are important to the research question that are missing, raising some critical questions about the thoroughness of the search and inclusion process.

Table 2 provides some details about the nature of the findings, but it is difficult to extrapolate some of them into specific practice behaviors. A more detailed reporting of the implications for practice, as it was reported by the study authors, would be helpful.

The findings from section 3 largely mirror what is known from navigation research with populations other than EDCs. It would be helpful to distinguish the unique barriers, facilitators, and interventions for EDC's and describe how they are similar or different to non EDC's. As it reads currently the strategies for working with EDC's appear to be universal. The section might also be better organized into sub themes within each of the 3 categories of findings to make the presentation of information more interpretable. For example, what are some indigenous, veteran, orr LGBTQ specific methods to promote service access? What unique barriers to they experience that differ from non EDC populations? works within or across populations? What are the strengths and haps gaps within the research for each EDC that was studies?

Reviewer #2: Dear Authors,

Thank you for the opportunity to review this important contribution to the literature on health navigation programs that support EDCs. This scoping review addresses an important and timely gap in the mental health and addiction (MHA) navigation literature by mapping approaches and practices used to support Equity‑Deserving Communities (EDCs). The review’s focus on populations that experience structural barriers to care—including low‑income individuals, racial and ethnic minorities, Indigenous Peoples, newcomers, people with disabilities, and members of 2SLGBTQI+ communities—aligns well with the scope of PLOS ONE Mental Health and current priorities in equitable mental health service delivery.

The manuscript is clearly written and methodologically transparent. The authors provide a detailed description of their search strategy and analytic procedures, follow Arksey and O’Malley and JBI guidance for scoping reviews, and strengthen rigor through protocol registration with the Open Science Framework. The purpose of the study and guiding research question are clearly articulated and well justified.

Summary of Strengths

Clear articulation of the study rationale and equity focus

Transparent and reproducible scoping review methodology

Protocol registration enhances credibility

Comprehensive descriptive mapping of MHA navigation approaches

Specific Comments and Suggestions

Conceptual Citation

The manuscript references Freeman’s conceptualization of navigation early in the introduction (lines 86–88). Including an explicit citation at this point would strengthen the conceptual grounding of the review and situate it more firmly within the foundational navigation literature.

Date Range Justification

A brief rationale for the decision to include studies published from 2014 onward would improve transparency. Clarifying whether this reflects shifts in MHA navigation practice, policy developments, or increased attention to equity‑oriented navigation models would be helpful.

Presentation of Included Studies

The ordering of studies in Table 1 is not immediately clear. A short explanation of the organizing logic (e.g., chronological, geographic, or thematic) would improve clarity.

One included source is described as a narrative review (p. 9). As narrative reviews are not typically considered primary sources within Arksey and O’Malley or JBI scoping review methodologies, the authors may wish to reconsider its inclusion or briefly justify its methodological fit within the data corpus.

Framing of Findings

Several sections of the findings include interpretive or prescriptive language that appears more appropriate for the Discussion or Implications sections. For example, section 3.2.2 (line 263) begins with “Therefore, programs should…,” which reads as a recommendation rather than a descriptive synthesis. Reframing results sections to remain primarily descriptive, while reserving recommendations for later sections, would strengthen alignment with scoping review conventions.

Theoretical Orientation and Positionality

Given the strong equity focus of the review, a brief theoretical framing and/or positionality statement may help orient readers to the analytic lens informing the interpretation of the literature. This could further enhance transparency and contextual understanding.

Generalizability and Knowledge Claims

The discussion of limited generalizability to global populations (p. 31, lines 456–457) may not be necessary. The review is primarily descriptive in nature and does not appear to adopt a positivist orientation or make population‑level causal claims. Emphasizing transferability—rather than generalizability—may be more appropriate for a scoping review of this kind and more consistent with qualitative and equity‑oriented synthesis standards.

Attention to Quantitative Evidence

The review does not appear to attend to quantitative findings or their integration into the analysis. Clarifying whether quantitative evidence was encountered but not synthesized, or explicitly noting that the review prioritized qualitative and descriptive data, would improve methodological transparency.

Overall Recommendation

This manuscript presents a valuable and well‑executed scoping review that contributes meaningfully to understanding how MHA navigation programs support equity‑deserving communities. The suggested revisions are primarily intended to clarify methodological decisions and strengthen alignment with scoping review reporting and interpretive conventions.

**********

6. PLOS authors have the option to publish the peer review history of their article (what does this mean?). If published, this will include your full peer review and any attached files.

**Do you want your identity to be public for this peer review?**  If you choose “no”, your identity will remain anonymous but your review may still be made public.

For information about this choice, including consent withdrawal, please see our Privacy Policy.

Reviewer #1: No

Reviewer #2: No

**********

[NOTE: If reviewer comments were submitted as an attachment file, they will be attached to this email and accessible via the submission site. Please log into your account, locate the manuscript record, and check for the action link "View Attachments". If this link does not appear, there are no attachment files.]

-->Figure Resubmissions:

While revising your submission, we strongly recommend that you use PLOS’s NAAS tool (https://ngplosjournals.pagemajik.ai/artanalysis) to test your figure files. NAAS can convert your figure files to the TIFF file type and meet basic requirements (such as print size, resolution), or provide you with a report on issues that do not meet our requirements and that NAAS cannot fix.

After uploading your figures to PLOS’s NAAS tool - https://ngplosjournals.pagemajik.ai/artanalysis, NAAS will process the files provided and display the results in the "Uploaded Files" section of the page as the processing is complete. If the uploaded figures meet our requirements (or NAAS is able to fix the files to meet our requirements), the figure will be marked as "fixed" above. If NAAS is unable to fix the files, a red "failed" label will appear above. When NAAS has confirmed that the figure files meet our requirements, please download the file via the download option, and include these NAAS processed figure files when submitting your revised manuscript.

# Author comment: Author response to Decision Letter 1

**DOI:** 10.1371/journal.pmen.0000557.r002

9 May 2026

Attachment Submitted filename: 20260508 PLOS Mental Health_Response.docx

# Peer review: Decision Letter 1

**DOI:** 10.1371/journal.pmen.0000557.r003

**Authors:** Karli Montague-Cardoso, Karli Montague-Cardoso

2 Jun 2026

Pathways to inclusion: A scoping review exploring equity, diversity, and inclusion in mental health and addictions patient navigation programs

PMEN-D-26-00024R1

Dear Dr Markoulakis,

We are pleased to inform you that your manuscript 'Pathways to inclusion: A scoping review exploring equity, diversity, and inclusion in mental health and addictions patient navigation programs' has been provisionally accepted for publication in PLOS Mental Health.

Before your manuscript can be formally accepted you will need to complete some formatting changes, which you will receive in a follow up email. A member of our team will be in touch with a set of requests.

Please note that your manuscript will not be scheduled for publication until you have made the required changes, so a swift response is appreciated.

IMPORTANT: The editorial review process is now complete. PLOS will only permit corrections to spelling, formatting or significant scientific errors from this point onwards. Requests for major changes, or any which affect the scientific understanding of your work, will cause delays to the publication date of your manuscript.

If your institution or institutions have a press office, please notify them about your upcoming paper to help maximize its impact. If they'll be preparing press materials, please inform our press team as soon as possible -- no later than 48 hours after receiving the formal acceptance. Your manuscript will remain under strict press embargo until 2 pm Eastern Time on the date of publication. For more information, please contact mentalhealth@plos.org.

Thank you again for supporting Open Access publishing; we are looking forward to publishing your work in PLOS Mental Health.

Best regards,

Karli Montague-Cardoso

Staff Editor

PLOS Mental Health

***********************************************************

Reviewer Comments (if any, and for reference):

Reviewer's Responses to Questions

**Comments to the Author**

1. If the authors have adequately addressed your comments raised in a previous round of review and you feel that this manuscript is now acceptable for publication, you may indicate that here to bypass the “Comments to the Author” section, enter your conflict of interest statement in the “Confidential to Editor” section, and submit your "Accept" recommendation.

Reviewer #2: All comments have been addressed

**********

2. Does this manuscript meet PLOS Mental Health’s publication criteria? Is the manuscript technically sound, and do the data support the conclusions? The manuscript must describe methodologically and ethically rigorous research with conclusions that are appropriately drawn based on the data presented.

Reviewer #2: Yes

**********

3. Has the statistical analysis been performed appropriately and rigorously?

Reviewer #2: N/A

**********

4. Have the authors made all data underlying the findings in their manuscript fully available (please refer to the Data Availability Statement at the start of the manuscript PDF file)?

The PLOS Data policy requires authors to make all data underlying the findings described in their manuscript fully available without restriction, with rare exception. The data should be provided as part of the manuscript or its supporting information, or deposited to a public repository. For example, in addition to summary statistics, the data points behind means, medians and variance measures should be available. If there are restrictions on publicly sharing data—e.g. participant privacy or use of data from a third party—those must be specified.

Reviewer #2: Yes

**********

5. Is the manuscript presented in an intelligible fashion and written in standard English?

PLOS Mental Health does not copyedit accepted manuscripts, so the language in submitted articles must be clear, correct, and unambiguous. Any typographical or grammatical errors should be corrected at revision, so please note any specific errors here.

Reviewer #2: Yes

**********

6. Review Comments to the Author

Please use the space provided to explain your answers to the questions above. You may also include additional comments for the author, including concerns about dual publication, research ethics, or publication ethics. (Please upload your review as an attachment if it exceeds 20,000 characters)

Reviewer #2: (No Response)

**********

7. PLOS authors have the option to publish the peer review history of their article (what does this mean?). If published, this will include your full peer review and any attached files.

**Do you want your identity to be public for this peer review?**  If you choose “no”, your identity will remain anonymous but your review may still be made public.

For information about this choice, including consent withdrawal, please see our Privacy Policy.

Reviewer #2: No

**********
